# Prostate Specific Membrane Antigen Expression in a Syngeneic Breast Cancer Mouse Model

**DOI:** 10.1007/s11307-024-01920-2

**Published:** 2024-05-17

**Authors:** Aditi A. Shirke, Jing Wang, Gopolakrishnan Ramamurthy, Arpan Mahanty, Ethan Walker, Lifang Zhang, Abhiram Panigrahi, Xinning Wang, James P. Basilion

**Affiliations:** 1 Department of Biomedical Engineering, Case Western Reserve University, 11100 Euclid Ave, Wearn Building B-49, Cleveland, OH 44106 USA; 2 Department of Radiology, Case Western Reserve University, 11100 Euclid Ave, Wearn Building B-49, Cleveland, OH 44106 USA

**Keywords:** Animal model supporting targeted imaging, Breast cancer, Cancer biomarker, Molecular imaging, Molecular imaging contrast agents, Prostate cancer, Prostate specific membrane antigen expression, Triple negative breast cancer

## Abstract

**Purpose:**

Prostate specific membrane antigen (PSMA) has been studied in human breast cancer (BCa) biopsies, however, lack of data on PSMA expression in mouse models impedes development of PSMA-targeted therapies, particularly in improving breast conserving surgery (BCS) margins. This study aimed to validate and characterize the expression of PSMA in murine BCa models, demonstrating that PSMA can be utilized to improve therapies and imaging techniques.

**Methods:**

Murine triple negative breast cancer 4T1 cells, and human cell lines, MDA-MB-231, MDA-MB-468, implanted into the mammary fat pads of BALB/c mice, were imaged by our PSMA targeted theranostic agent, PSMA-1-Pc413, and tumor to background ratios (TBR) were calculated to validate selective uptake. Immunohistochemistry was used to correlate PSMA expression in relation to CD31, an endothelial cell biomarker highlighting neovasculature. PSMA expression was also quantified by Reverse Transcriptase Polymerase Chain Reaction (RT-PCR).

**Results:**

Accumulation of PSMA-1-Pc413 was observed in 4T1 primary tumors and associated metastases. Average TBR of 4T1 tumors were calculated to be greater than 1.5—ratio at which tumor tissues can be distinguished from normal structures—at peak accumulation with the signal intensity in 4T1 tumors comparable to that in high PSMA expressing PC3-pip tumors. Extraction of 4T1 tumors and lung metastases followed by RT-PCR analysis and PSMA-CD31 co-staining shows that PSMA is consistently localized on tumor neovasculature with no expression in tumor cells and surrounding normal tissues.

**Conclusion:**

The selective uptake of PSMA-1-Pc413 in these cancer tissues as well as the characterization and validation of PSMA expression on neovasculature in this syngeneic 4T1 model emphasizes their potential for advancements in targeted therapies and imaging techniques for BCa. PSMA holds great promise as an oncogenic target for BCa and its associated metastases.

**Supplementary Information:**

The online version contains supplementary material available at 10.1007/s11307-024-01920-2.

## Introduction

Breast cancer (BCa), the second most prominent of all cancers, is estimated to have upwards of 280,000 new cases in the United States and 2.3 million cases worldwide in 2023 [1]. Medical imaging and diagnosis advances, for example mammography and digital breast tomosynthesis (DBT), allow cancer detection at an earlier stage, thus around 64% of cases are localized to a primary tumor site [2] and women with stages 1–3 undergo breast conserving surgery (BCS) as the primary treatment. However, incomplete resection occurs in 20%-60% of BCS leading to repeated procedures with further stress and financial burden on the patients [3–5]. Additionally, local recurrence occurs in 5%-16% of patients with pathologically clean margins, an indication of “surgically missed” cancerous tissues, indicating that regions of tumor were not sampled during the pathological analysis of excised tissues [6–8]. This demonstrates that there is an unmet clinical need for methods that identify and destroy cancerous tissues in the margins of BCS specimens through targeting theranostic agents to oncogenic biomarkers overexpressed in these tissues.

Prostate specific membrane antigen (PSMA) is a type II transmembrane protein consisting of 750 amino acids, and its overexpression in prostate cancer (PCa) compared to healthy prostate tissues is well-established [9]. PSMA has a unique internalization motif MXXXL and is reported to have a robust baseline internalization rate of 60% of its surface PSMA in 2 h [9, 10]. PSMA holds several vital enzymatic activities, for example, folate hydrolase activity, so PSMA also goes by the term folate hydrolase-1 (FOLH1) [11]. FOLH-1 enzymes and other important molecular activities lead to increased angiogenesis and cell proliferation in tissues and cancers, which further increase expression of PSMA in advanced stages of the disease [12–16].

PSMA and its respective genetic sequences and transcripts have been thoroughly studied due to its potential use as a biomarker. Research has linked PSMA expression to the FOLH1 gene, examining how expression levels affect outcomes, supporting FOLH1 gene expression as an indicator of PSMA protein expression [17–21]. Mouse PSMA expression shows around 86%-91% similarity to human PSMA expression with both variants possessing similar biomolecular activities and function [11, 22]. However, it is important to acknowledge that the translation of gene expression to protein levels may not be completely mapped, especially in varying cancer models across a large population. Further investigation into FOLH1 and PSMA expression in human cancers is crucial for understanding expression patterns and the connection between FOLH1 gene expression and PSMA protein levels. Through matching PSMA expression, diagnosis, and treatment in previous patient case studies, we can use this information to potentially use PSMA as a prognostic indicator in clinical settings [23–26]. RT-PCR, immunohistochemistry (IHC) and other characterization techniques have further confirmed this overexpression in other solid cancers.

While PSMA overexpression is primarily localized on the cells in PCa, literature shows PSMA being overexpressed primarily on the neovasculature of multiple solid human tumors, including BCa, along with some overexpression on tumor cells themselves [27–30]. Multiple studies have analyzed human BCa samples and quantified PSMA expression in vessels, tumors, metastases and healthy tissue as reviewed by Clara Unger et al*.* [31]. Moreover, there is no endothelial expression of PSMA under normal physiological conditions which makes it a favorable target for imaging and treating solid cancers [32, 33]. It has been shown that 60%—74% of human primary breast tumors express PSMA [34]. From these studies, significant overexpression of PSMA in the neovasculature of human BCa tumors is evident. Further, there is a correlation of significantly higher PSMA expression between metastatic sites, for example in the brain, and primary tumors, which has been demonstrated *in vivo* in human samples [35]. BCa tumors that possess greater than 50% of PSMA-positive vasculature are significantly larger, of higher histological grade, more proliferative, and more likely to be metastatic [34]. In addition, studies have revealed that free-circulating BCa cells in human patients displaying positive PSMA expression, often indicated a less favorable prognosis for these patients [36].

The lack of established *in vitro* or *in vivo* experimental mouse models that specifically represent PSMA-expressing neovasculature has impeded the development of imaging and therapeutic approaches using PSMA-targeting for other tumor models including BCa. There are a few reported *nu/nu* animal models for vascular PSMA expression in response to tumor challenge, including human triple negative breast cancer (TNBC) cell lines MB-MDA-231 and MB-MDA-468 and estrogen receptor expressing (ER +) BCa models [37]. Using conventional nude mouse models in cancer studies presents a limitation due to their incomplete immune systems, making it difficult to explore immune responses. Consequently, our lab has turned to syngeneic mouse models to delve into understanding how the tumor immune microenvironment (TIME) and the recruitment of immune cell precursors affect BCa therapy outcomes in mice [38].

PSMA-1 is a highly specific PSMA ligand that we have previously developed in our lab, (Suppl. Fig. S1 in the electronic supplemental material (ESM)). It has a binding affinity fivefold higher than the parent PSMA ligand, which we have successfully used to visualize tumors of multiple human prostate cancer cell lines [39, 40]. A few other examples of our PSMA-1 targeted agents include PSMA-1-Cy5.5 for fluorescence imaging; theranostic agents PSMA-1-Pc413 and PSMA-1-IR700 for photodynamic therapy (PDT) and PSMA-1 targeted gold nanoparticles for radiotherapy sensitization [41–43]. We utilized PSMA-1-Pc413 to successfully identify cancerous tissues and metastases, enabling live fluorescence image guided surgery (FIGS) [39].

In this study, we have utilized an orthotopic syngeneic mouse model of BCa and have confirmed that PSMA is overexpressed on the neovasculature of 4T1 BCa in athymic BALB/c mice as well as immunocompetent BALB/c mice. We additionally validate the potential for PSMA to be used as a BCa biomarker in these models by visualizing these tumors *in vivo* with our highly selective PSMA targeted fluorescence imaging agents [39]. The studies here open up the possibility of utilizing an immunocompetent mouse model to study PSMA expression in BCa neovasculature as a biomarker for PSMA-targeted therapeutics.

## Materials and Methods

### UCSC Xena—Data Processing and Statistical Analysis

Data including, RNA-seq, clinical diagnoses, pathological markers and patient survival, were obtained from the UCSC Xena Portal (https://xena.ucsc.edu/) which accesses The Cancer Genome Atlas (TCGA), Genotype-Tissue Expression (GTEx) and other large, publicly available cancer data consortiums. The data was sorted, and FOLH1 gene expression data were extracted by creating visual spreadsheets for the desired cohort groups, (Suppl. Table S1, see ESM) e.g., sample type (primary tumor), gender (Female for BCa), and analyzed using R (Foundation for Statistical Computing) and visualized through GraphPad Prism (GraphPad Software, San Diego, CA, USA).

### Cell Culture

PSMA expressing PC3-Pip and PSMA negative PC3-Flu human PCa cells, as well as human BCa MDA-MB-231 and MDA-MB-468, were maintained in RPMI1640 medium with 10% fetal bovine serum (FBS), 1% penicillin–streptomycin solution (PS) for a maximum of 6 passages before discarding. 4T1 cells were maintained in Dulbecco’s modified eagle medium (DMEM) GlutaMAX (high glucose) media with 10% FBS, 1% PS for a maximum of 8 passages before discarding. Mycoplasma testing was last performed in February 2023 and all parent and subsequent cell lines are Myoplasm free.

PC3-Pip cells are human prostate cancer PC3 cells that have been transformed to have high human PSMA expression, and PC3-Flu cells are PC3 cells transformed with empty vector only and do not express PSMA. We have previously demonstrated that PC3-Pip cells show similar expression levels compared to lymph node carcinoma of the prostate (LNCaP) cells through western blot and other *in vitro* studies, illustrated in Fig. S7 *of* our previous publication, *Wang, X., *et al*., Photodynamic Therapy Is an Effective Adjuvant Therapy for Image-Guided Surgery in Prostate Cancer. Cancer Res, 2020. 80(2): p. 156–162.* [39]. PC3-Pip cells have a faster *in vivo* growth rate compared to LNCaP allowing for more favorable experimental timelines.

### Synthesis of PSMA-1-Alexa488

PSMA-1-Alexa488 was synthesized using a similar method as synthesis of PSMA-1-IR800 and PSMA-1-Cy5.5 [44]. PSMA-1 [45] 1.0 mg (0.92 µmol) was dissolved in 0.5 mL PBS, pH 7.5, to which, Alexa488 NHS ester 1.0 mg (1.6 µmol) (Thermo Fisher) in 0.5 mL PBS was added. The mixture was stirred in the dark at room temperature for 24 h, then purified by semi-preparative High Performance Liquid Chromatography (HPLC), with a retention time at around 19 min. The structure of PSMA-1-Alexa488 was validated by mass spectrometry (MS): C67H94N12O30S2, calculated: 1610; found: 1632 (M = Na) (Fig. S2). Additionally, PSMA specificity and fluorescence spectra of the probe was confirmed through *in vitro* studies highlighting specific uptake of PSMA-1-Alexa488 in PSMA positive PC3-pip prostate cancer cells before and after blocking with excess amount of (S)-2-(3-((S)-5-amino-1-carboxypentyl)ureido)pentanedioic acid (ZJ24) to block the PSMA receptor binding and demonstrate selective probe uptake as carried out in previous studies (Fig. S3) [45–47].

### Histological Staining and Microscopy

Animal experiments were approved by the University Institutional Animal Care and Use Committee (IACUC), Protocol Number: 2015–0033.

To obtain orthotopic murine 4T1 tumors, six- to eight-week-old immunocompetent female BALB/C and athymic female BALB/C nude mice were obtained from The Jackson Laboratory and implanted with 4T1 cells in the mammary fat pads. Tumor and metastases formation during preliminary experiments were assessed by bioluminescence imaging to form these 4T1 tumor models (Fig. S4, see ESM) and it was found that mice received 10,000 cells in 100 μL of phosphate-buffered saline (PBS) showed the fastest growth rate and the earliest metastasis formation, therefore this cell number was selected to implant 4T1 tumors.

To obtain orthotopic human BCa cells, 1 × 10^6^ cells of either MDA-MB-468 or MDA-MB-231 cells, in 100 µL of PBS, were implanted in immunocompromised female BALB/c mice.

To obtain flank prostate tumors, six- to eight-week-old male BALB/C nude mice were implanted subcutaneously with 1 × 10^6^ of PC3-Pip or PC3-Flu cells, in a total 100 μL 1:1 ratio of PBS and Matrigel, on the right dorsum.

Tumors were allowed to grow to ~ 150–200 mm^3^, measured by calipers. Tumor volume was calculated using width^2^/length/2 ensuring width and length orientation remained consistent while measuring the tumors. 4T1 tumors reached this size at around 10–12 days while PC3-Pip, PC3-Flu, MDA-MB-231 and MDA-MB-468 reached this size around 14–16 days. Mice were euthanized, tissues were explanted from mice and embedded in optimal cutting temperature (OCT) compound. For PSMA-CD31 staining, 12 μm thick tissues on slides were co-incubated with PSMA-1-Alexa488, synthesized in the lab, and anti-CD31 antibody (Abcam, anti-CD31, #ab28364) overnight at 4℃. After washing, tissues were incubated with goat anti-rabbit AlexaFluor594 (ThermoFisher, R37117).

For blocking studies, the adjacent slices from the same region were co-incubated with 20 nmol/L PSMA-1-Alexa488 and tenfold excess amount of ZJ24 to block the PSMA receptor binding and demonstrate selective probe uptake as carried out in previous studies.

All slides were then incubated with Hoechst solution and sealed for fluorescence microscopy (Hoechst 33,342, trihydrochloride, trihydrate—10 mg/mL solution in water, Thermo Fisher). For commercially available PSMA primary antibody staining, we used Prostate Specific Membrane Antigen (D7I8E) XP® rabbit mAb #12,815 (Cell Signaling Technology) again paired with AlexaFluor594 (pseudo colored magenta instead of red). Standard hematoxylin and eosin (H&E) staining protocols were used to stain the tissues as described below.

Fluorescence images were captured with Leica-DM4000B microscope and the corresponding QCapturePro-7 software. PSMA-CD31 colocalization analysis and H&E images were captured through the Keyence Microscopy system (BZ-X Series).

To carry out colocalization analysis on these tissues, we ensured that all images taken for this purpose were under the same imaging settings. This would confirm that signals we observed were positive and true and didn’t affect further statistical processing. Altering these between images would alter pixel values and therefore affect auto-thresholding through Fiji ImageJ JACoP. Alexa 594 stains were visualized using the TX2 filter (BP 560/40 nm) at 700 ms, PSMA-1-Alexa488 visualized on the L5 filter (BP 480/40 nm) at 1200 ms and Hoechst was visualized using the A4 Filter (BP 360/40 nm) at 100 ms. Other imaging settings, such as gain (4) and offset (50), remained constant. Images were saved and analyzed as 12-bit image files.

### H&E Staining Protocol

Frozen sections were thawed and fixed with 4% paraformaldehyde for 10 min accompanied by washing and removing OCT for 30 min in PBS. Tissues were stained in modified Harris’ hematoxylin (HX; Sigma-Aldrich, 3050 Spruce Street, Saint Louis, MO 63103, USA; # HHS16) for 6 min and then washed under tap water. Tissues were placed in 1% acid-alcohol (Sigma-Aldrich, USA; # A3179) for 1–3 s to remove excess stain and further washed with Scott's tap-water substitute (Sigma-Aldrich, USA; # S5134) to regain blue nuclei color. Tissue sections were stained with 1% eosin (Sigma-Aldrich, USA; #HT110116) solution for 2 min, differentiated in 70% alcohol, and promptly rinsed in water. Tissue sections were dehydrated in alcohol baths in an increased concentration manner, 70% > 95% > 100% alcohol for 10 s per bath. Finally, sections were placed in 3 xylene baths (Thermo Fisher Scientific, USA; #6601) prior to mount in Shandon-Mount Mounting media (Thermo Fisher Scientific, USA).

### Statistical Analysis

To extensively analyze PSMA-CD31 colocalization, primary tumors and lung metastases from 6 animals were approximately divided into fifths, and 3 pieces of tissue were taken from throughout these sections. A total of 90 samples were scanned per tissue type for fluorescence and H&E staining. Analyzing entire slices, through fluorescence microscopy we quantified intensity of positive PSMA (green) and CD31 (magenta) signals and used Fiji ImageJ plugin, JACoP (Suppl. Fig. S5, see ESM**)** [48], to apply a threshold and quantify this colocalization. Manders coefficients (MC), which demonstrates percentage overlap of PSMA on CD31, and Pearson correlation coefficient (PCC) were determined to demonstrate the nature of the relationship of the two markers *i.e.* level of positive correlation.

The average PCC was obtained by calculating Fisher's Z values for the individual coefficients, averaging them, and then reverse-transforming the average value. For the averages of MC, geometric mean methods were used, involving the nth root of the product of values in the dataset, where the product of values is rooted to the dataset's size.

For arriving at the sample size to allow us to determine if results are significant for this study, the software G Power [49] was used and the following parameters were used as inputs: Statistical test:—Wilcoxon-Mann–Whitney test (two groups), Direction of effect:- two tailed, Power:- 95%, Type 1 error:- 5%, Effect Size: 0.61 (based on a pilot study) which provided the sample size of 75. From our entire dataset, 75 data points of corresponding PCC and MC values were randomly chosen for both primary tumor and lung metastases tissues.

### *In Vivo* Biodistribution and Accumulation Studies

For 4T1 tumor xenografts, six- to eight-week-old female BALB/c mice were orthotopically implanted with 10,000 4T1-Luc or 4T1 cells via injecting these cells into the mammary fat pads. Similarly, for MDA-MB-231 or MDA-MB-468 tumor xenografts, female athymic BALB/c mice were orthotopically implanted with 1 × 10^6^ human BCa cells, either MDA-MB-231 or MDA-MB-468. To achieve prostate tumor xenografts, six- to eight-week-old male athymic BALB/c mice were implanted with 1 × 10^6^ human PCa cells in the right dorsum.

The tumors were allowed to grow up to ~ 100–200 mm^3^, measured by calipers, after which mice received 0.5 mg/kg PSMA-1-Pc413 via tail-vein. The mice were imaged at various time points till 72 h post injection using the Maestro *In Vivo* Imaging System (Perkin Elmer)—yellow filter was used to visualize PSMA-1-Pc413 signal with excitation 575–605 nm and a longpass 645 nm emission filter. To confirm tumor and metastases, bioluminescence images were taken using IVIS Spectrum *In Vivo* Imaging System (Perkin Elmer) [50] for which mice received an intraperitoneal (IP) injection of D-luciferin (Syd Labs, Boston).

Tumor background ratio (TBR) is a method of quantifying the efficacy of uptake of imaging probes in cancerous tissues compared to normal structures and their “background signal”. This provides a measure of how cancerous tissues will be visualized compared to normal structures especially when considering probes for live imaging during surgical processes. This measure has been validated in multiple human cancer models including breast cancer [51, 52]. For these studies, the background is determined using the spleen when organs are extracted since it is relatively unaffected from metastases formation in our models. For liver imaging, TBR is determined by creating a region of interest (ROI) on the abdomen (skin/fur) and taking this as a background signal. Additionally, this signal value is used as background whilst normalizing values to background.

### RT-PCR of Tumor Tissues

4T1 tumors were grown orthotopically in BALB/c mice and allowed to grow to ~ 200 mm^3^, measured using calipers, at which size metastases began to develop infiltrating into the lungs or flank muscles, as described from previous studies, (Suppl. Fig. S4, see ESM). For this study, tumors and lungs with varying degrees of metastases were extracted from 4 mice orthotopically inoculated with 4T1 cells and healthy muscles and lungs were extracted from 4 healthy mice. These tumors and lung tissues were then extracted, snap frozen and saved in -80℃. Tissue samples were then pulverized, homogenized and RNA was extracted using the miRNeasy Mini Kit (Qiagen, 217,004). Total RNA concentration was then measured using NanoDrop Spectrophotometer (Thermo Fisher). We then prepared cDNA from our samples through the High-Capacity RNA-to-cDNA kit (Applied Biosystems, 4,387,406). RT-PCR was then carried out using TaqMan Reagents – mouse GAPDH and mouse FOLH1, TaqMan Universal Master Mix II, with the synthesized cDNA. The RT-PCR was run on StepOne Device and analyzed through its software (Thermo Fisher) with triplicates from each tissue sample as well as triplicates of the GAPDH.

## Results

### PSMA Expression on Human BCa *in vivo*

To confirm PSMA gene, denoted as FOLH1, expression in various non-prostatic human primary tumors, we analyzed ribonucleic acid sequencing (RNA-Seq) data from the University of California Santa Cruz (UCSC) Xena database for multiple solid cancers and selected the cohort based on FOLH1 expression in primary tumor tissues only for each cancer type (Suppl. Table. S1, see ESM). By analyzing The Cancer Genome Atlas (TCGA) RNA-Seq data, it was observed that FOLH1 is positively expressed in multiple solid cancers such as BCa, pancreatic cancer, and ovarian cancer, which means PSMA is also positively expressed in these cancers (Suppl. Fig. S6, see ESM). This broader pattern of PSMA expression in varying cancers highlights its potential importance as a target for diagnostics and therapeutics in a variety of solid tumors, extending beyond its well-known role in PCa.

### PSMA Expression on Human BCa *in vivo*

**Fig. 1:**
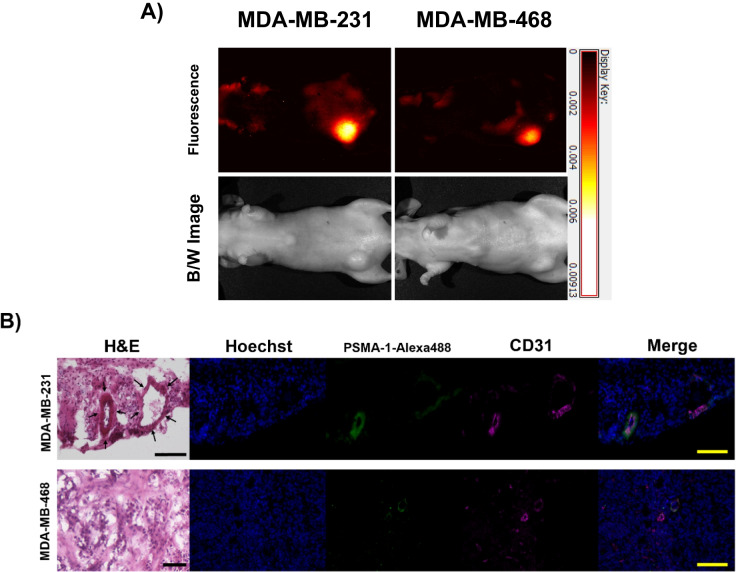
PSMA expression on human, MDA-MB-468 and MDA-MB-231, breast cancer neovasculature. **(A)** Fluorescence Imaging of MDA-MB-231 (L) and MDA-MB-468 (R) mice injected with PSMA-1-Pc413 via tail-vein injection. TBR values: MDA-MB-231: 5.35; MDA-MB-468: 5.21. **(B)** PSMA-CD31 staining in human breast cancer MDA-MB-231 (1st row) and MDA-MB-468 (2nd row) derived from Athymic (i.e., Nude/Nu) female BALB/c mice as above. All images were captured at 20x. Yellow and black bars indicate 100 µm distance

To study PSMA-CD31 colocalization *in vivo*, we inoculated female athymic BALB/c mice with two human TNBC cell lines, MDA-MB-231 and MDA-MB-468. Intravenous (IV) administration of PSMA-1-Pc413 followed by whole animal imaging showed positive accumulation of PSMA-1-Pc413 within the tumor tissues with good contrast compared to normal surrounding tissues Fig. 1A. Upon quantifying the fluorescence signals, comparative signal strength was evident (Suppl. Fig. S7, see ESM). Immunohistostaining of the tumor tissues showed a positive correlation of PSMA (green) and CD31 (magenta) in the merged image (white) signifying PSMA colocalization on the neovasculature. Figure 1B. These data showed that PSMA is expressed on the tumor vasculature *in vivo* in these human breast cancer models. However, not all CD31 signals were associated with PSMA staining, suggesting only a subset of vasculature expressed PSMA. Additionally, in certain sections such as in the representative stains for MDA-MB-231—Fig. 1B top row – the signals for CD31 and PSMA do not show strong overlap within the neovasculature.

This is reflected in PCC and MC values for images in Fig. 1B. PCC is a widely used statistical measure that quantifies the strength and direction of the linear relationship between two variables and provides a number between -1 and 1, where 1 indicates a strong positive relationship, -1 indicates a strong negative relationship, and 0 indicates no relationship. Hence, along with identifying the presence or absence of correlation it also determines the exact level to which the variables are correlated thereby highlighting interdependencies. PCC has some limitations as it may not accurately capture relationships that are non-linear, and it does not distinguish between dependent and independent variables and hence cannot identify which variable is causing an influence. However, since the objective of this study is determining the existence of correlation, PCC is a sufficient method in analyzing tissues [53].

MC analysis, a widely used co-occurrence measure, gauges the percentage overlap between signals in two fluorescent channels, often applied in colocalization studies exploring biomarker interaction. However, it might inaccurately reflect the relationship if background noise is prevalent, being insensitive to signal-to-noise ratio yet responsive to out-of-focus signals. This limitation is addressed by applying thresholds to eliminate noise (Suppl. Fig. S5, see ESM). MC analysis yields a percentage overlap, where an MC value of 0.34 indicates 34% signal overlap in a data point [53].

MDA-MB-231 (top row): PCC = 0.147 and MC = 0.071 signifying low correlation and low overlap, however, we can confirm that both PSMA and CD31 are being expressed within the vasculature structure through H&E stains. This data can be inferred as a 7.1% overlap between PSMA and CD31. MDA-MB-468 (bottom row): PCC = 0.316, MC = 0.607 signifies a low correlation since very few vasculature structures express PSMA, however, this overlap is relatively high and provides a higher MC value, inferred 60.7% overlap (Suppl. Table S3, see ESM).

### PSMA is Overexpressed in Murine BCa Tumors on the Tumor Neovasculature

**Fig. 2:**
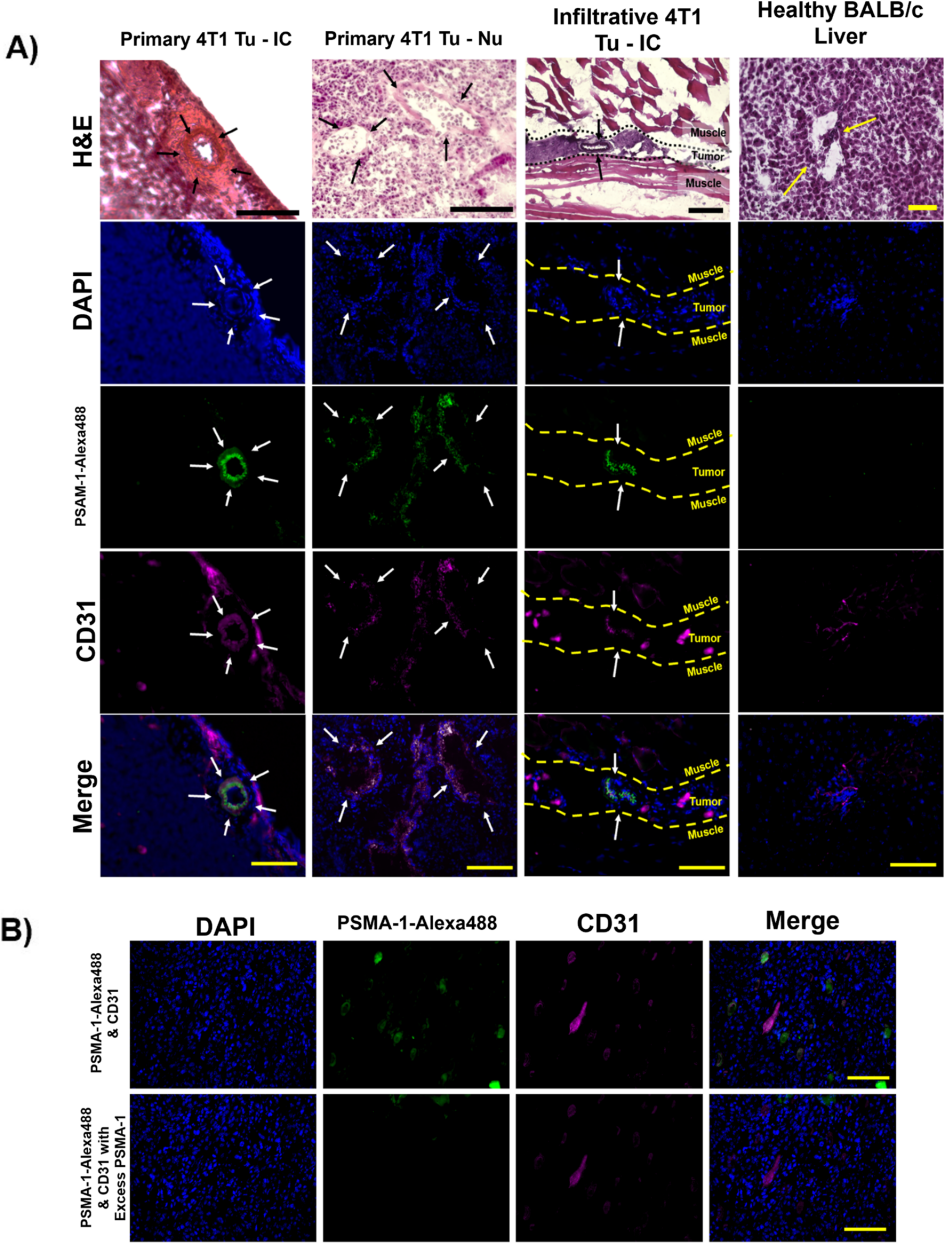
PSMA overexpression on murine, 4T1 primary tumor neovasculature. **(A)** H&E, and PSMA-CD31 staining using PSMA-1-Alexa488 (green) and CD31 (magenta). The merge in the last row shows overlap between DAPI, PSMA and CD31 staining. Slices were processed from 4T1 tumors extracted from 3 different female BALB/c mice. Column 1 shows a circular tumor vasculature in an immune competent BALB/c mouse, near the tumor margins, showing PSMA-CD31 colocalization (20 × magnification). Column 2 shows another vessel from an athymic BALB/c mouse, near the tumor core also showing PSMA-CD31 colocalization (20 × magnification). Column 3 shows a 4T1 tumor embedded into the muscle with the dashed lines highlighting the tumor tissue (20 × magnification). Column 4 shows blood vessels in healthy liver tissues from healthy female BALB/c mice (20 × magnification). Measurement Bars represent 100 μm. **(B)** Staining of tumor tissues to show DAPI staining (blue), PSMA (green) and CD31 (magenta). (TOP) The white in the “Merge” showing co-localization of PSMA-CD31 in the tumor tissues showing PSMA expression in 4T1 neovasculature. (BOTTOM) Addition of tenfold more PSMA-1 to block the receptors show specificity to PSMA. Images captured at 20 × magnification; measurement bars represent 100 μm

While the study above shows positive results for PSMA *in vivo* target in two human TNBC cell lines, these animal models were carried out in immune deficient, i.e., athymic or nude mice, and lack potential to carry out analyses on the immunological effects of treatments on these animals. The 4T1 cell line, derived from BALB/cfC3H mice, represents a TNBC subtype of BCa lacking estrogen receptor (ER), progesterone receptor (PR), and human endothelial growth factor (HER2) expression. This cell line closely resembles Stage IV and advanced basal phenotypes in human BCa with increased metastatic activity, primarily to the lungs, bones, liver, and brain, serving as a model to study BCa metastases [44]. We have characterized a syngeneic mouse breast tumor model wherein we inoculated murine 4T1 cells into the breast pads of female BALB/c mice. Tumors were allowed to grow to ~ 200 mm^3^ at which size metastases began to develop, infiltrating into the lungs or flank muscles as confirmed by bioluminescent imaging (Suppl. Fig. S4, see ESM). After frozen sectioning and carrying out IHC (Fig. 2A), we determined the expression of PSMA and CD31 in the primary and metastatic tumors. Column 1 shows IHC from a representative animal for primary 4T1 tumors inoculated into the breast pad of immunocompetent BALB/c mice; Column 2 shows similar data from 4T1 cells inoculated into nude immunodeficient mice; and Column 3 shows IHC for metastatic flank/bone tumors that resulted after orthotopic inoculation into female BALB/c mice. Column 4 shows IHC for healthy liver tissue that has been extracted from healthy BALB/c mice. For primary tumors implanted into both immunocompetent and nude mice, columns 1 and 2 respectively, there was moderate positive colocalization between PSMA and CD31 staining. Column 1: PCC = 0.534, MC = 0.381; Column 2: PCC = 0.627, MC = 0.541, inferring a 38% and 54% overlap between PSMA and CD31 staining (Suppl. Table S3, see ESM).

For metastasis to the leg, column 3, we observed the presence of PSMA colocalized on the neovasculature only in the tumor tissue and not in the healthy muscular tissue. While there are some staining artifacts visible, we believe this is a non-specific accumulation of Alexa Fluor 594 secondary antibody applied as any corresponding morphological structure that correlated with this pattern in the H&E stains was not observed. The merged images show the co-localization of the PSMA and CD31, PCC = 0.336, MC = 0.281.

Additionally, the liver tissue shows no PSMA staining. This data provides strong evidence that PSMA is overexpressed in the neovasculature of a syngeneic 4T1 tumor. Higher magnification of the IHC staining for PSMA and CD31 highlighted a notable correlation within the tumor neovasculature (Suppl. Fig. S8, see ESM). Interestingly, the PSMA expression displayed a distinctive staining pattern throughout the blood vessel with the CD31 vasculature expression.

To confirm PSMA specificity, we blocked the binding of PSMA-1-Alexa488 to PSMA with a tenfold excess of ZJ24, Fig. 2B. PSMA expression was once again found to be co-localized with CD31, indicating the presence of PSMA expression in tumor vasculature. After blocking with excess ZJ24 in adjacent slices within the same tumor sample, we detected no PSMA-1-Alexa488 signal (second row) while the CD31 signal remains largely unchanged Fig. 2B. This indicates specificity of PSMA-1-Alexa488 towards PSMA within the neovasculature as well as confirming the expression of PSMA in these tissues. In addition to PSMA-1-Alexa488, adjacent sections of the tumor tissue were also stained with anti-PSMA antibody and PSMA expression was again found on the tumor neovasculature, confirming PSMA expression in tumor vasculature (Suppl. Fig. S9, see ESM). While the tissues that were stained with primary PSMA Ab were not stained with CD31, similar staining patterns between the use of PSMA-1-Alexa488 and anti-PSMA Ab at the same area in adjacent sections validates the expression of PSMA on the neovasculature as well as the use of our probe for visualizing PSMA at a molecular level.

In contrast, high PSMA expressing human PCa cell line, PC3-pip cells, implanted into nude mice show high levels of PSMA expression on the tumors and not on the neovasculature, (Suppl. Fig. S10, see ESM), as was observed for the human BCa tumors, Fig. 1. PC3-Flu prostate cancer tumors, that show little to no expression of PSMA, were also stained for PSMA-CD31 with no positive staining visualized on the cells or neovasculature.

### PSMA -CD31 Colocalization Analysis

**Fig. 3:**
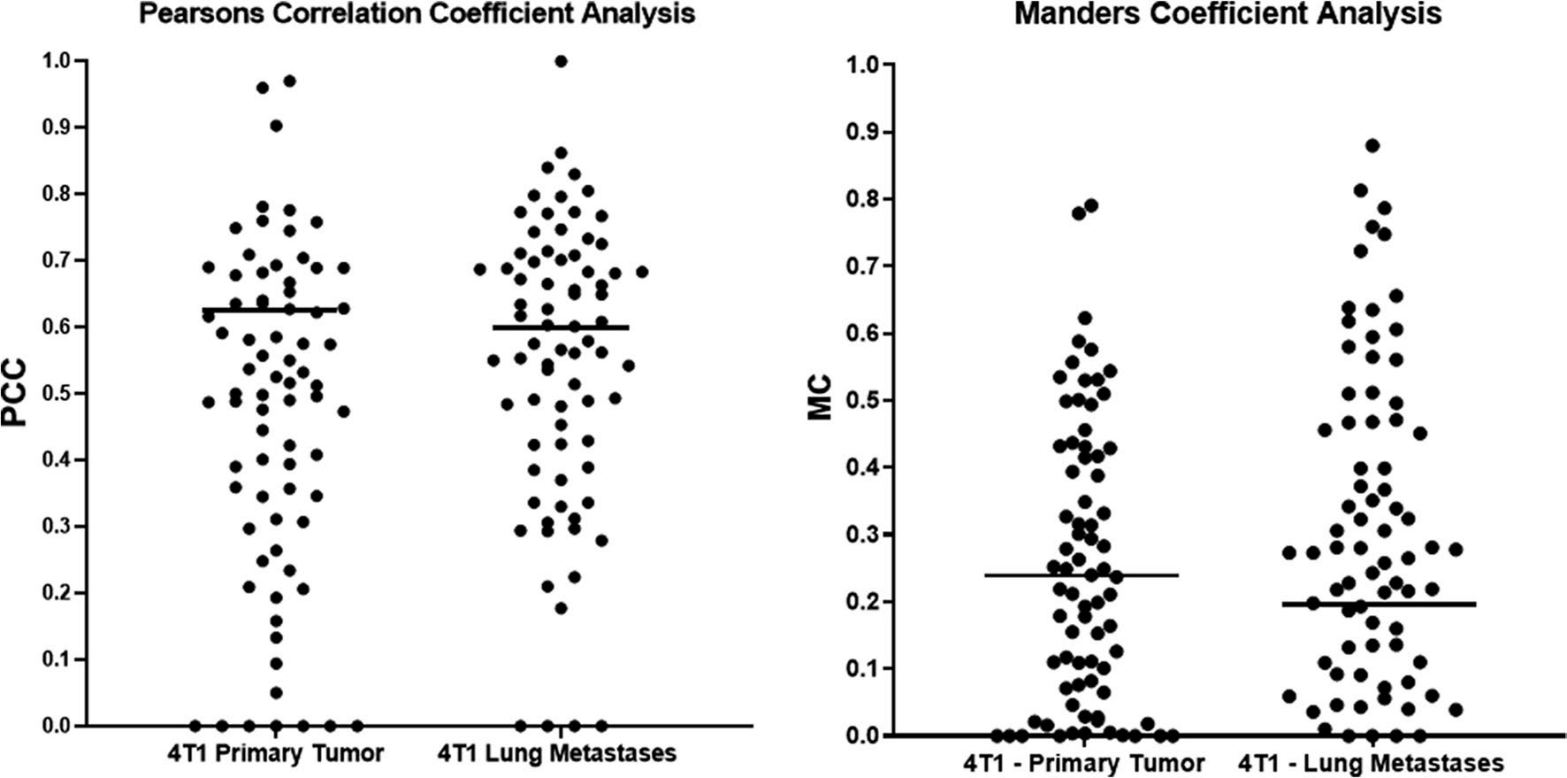
Quantification of PSMA-CD31 colocalization through PCC and MC. Carrying out a test on a wide range of tissue samples and quantifying both the extent of correlation through PCC and percentage overlap of PSMA over CD31 with MC. We can see that most tissue samples showed positive correlation and overlap indicating colocalization of PSMA-CD31. While carrying out a Fischer Transform Indicator with our Pearson values and quantifying the mean, we see mean PCC for the tumor and mean PCC for the Lung metastases are both > 0.5 which can be inferred that both tissues have a relatively high positive correlation between PSMA and CD31

For these studies, in total 90 4T1 tumor sections and 90 lung metastases sections were analyzed to get a quantitative assessment of PSMA-CD31 colocalization. For examining these 4T1 tumors and lung metastases tissues, we employed two methods to quantify this colocalization: the Pearson's correlation coefficient (PCC) and the Manders coefficient (MC), Fig. 3 [53], (Suppl. Table S3, see ESM).

PCC analysis of these 4T1 tumor tissues shows a strong positive correlation with most values being above 0 and the average PCC value, calculated through a Fisher Transform Indicator, are 0.610 for tumor tissues and 0.617 for lung metastases Fig. 3. Examples of positive PSMA expression and little to no PSMA expression with their corresponding PCC and MC values are reported in Suppl. Fig. S11 (see ESM). This signifies a strong correlation between the PSMA and CD31 signals in both types of tissues.

Analyzing colocalization in primary 4T1 tumors and lung metastases based on MC values, showed the geometric means for both sets of tissues were 0.200 in tumor tissues and 0.240 in lung metastases. This translates to a 24% and 20% overlap between PSMA-CD31 signals in tumors and lung metastases respectively.

Collectively, these findings strongly support that the expression of PSMA is positively correlated and localized predominantly on tumor neovasculature in both primary 4T1 tumors and their lung metastases. While CD31 highlights endothelial cells predominantly within the inner walls of the blood vessels, PSMA is expressed throughout the entire structure of the blood vessel and so while the expression shows significant positive correlation, the overlap between the two signals is not perfect as interpreted by the PCC and MC values.

### PSMA is Overexpressed in Murine BCa Lung Metastases on the Neovasculature

**Fig. 4:**
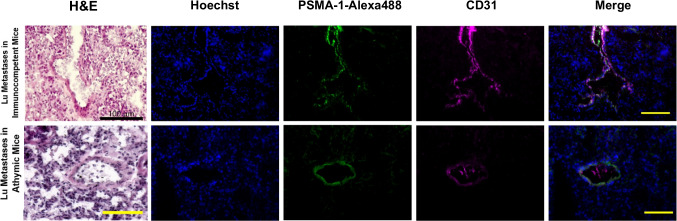
PSMA overexpression on murine, 4T1 lung metastases neovasculature. Staining of 4T1lung metastases in immunocompetent (top row) and Athymic/Nu BALB/c mice (bottom row) to show PSMA-1-Alexa488 (green) and CD31 (Magenta). The white in the Merge showing co-localization of PSMA-1-Alexa488 and CD31 in the metastatic tissues showing PSMA expression in 4T1 neovasculature. Scale bars are at 100 μm. TOP ROW: PCC = 0.711, MC = 0.801. BOTTOM ROW: PCC = 0.481, MC = 0.32

Lungs from mice implanted orthotopically with 4T1 tumors with significant metastasis to the lungs were stained using PSMA-1-Alexa488 and CD31. Similarly, to the primary tumors, PSMA expression was present on the neovasculature of the metastatic lung tumors Fig. 4. While the vasculature in the lung tumors is much smaller and more deformed compared to primary tumor tissues, clear colocalization, in white, of PSMA in these structures is visible. The expression of PSMA in the metastatic lung tumors was also confirmed by staining with anti-PSMA antibody (Suppl. Fig. S12, see ESM).

### Quantifying PSMA Gene Expression

**Fig. 5:**
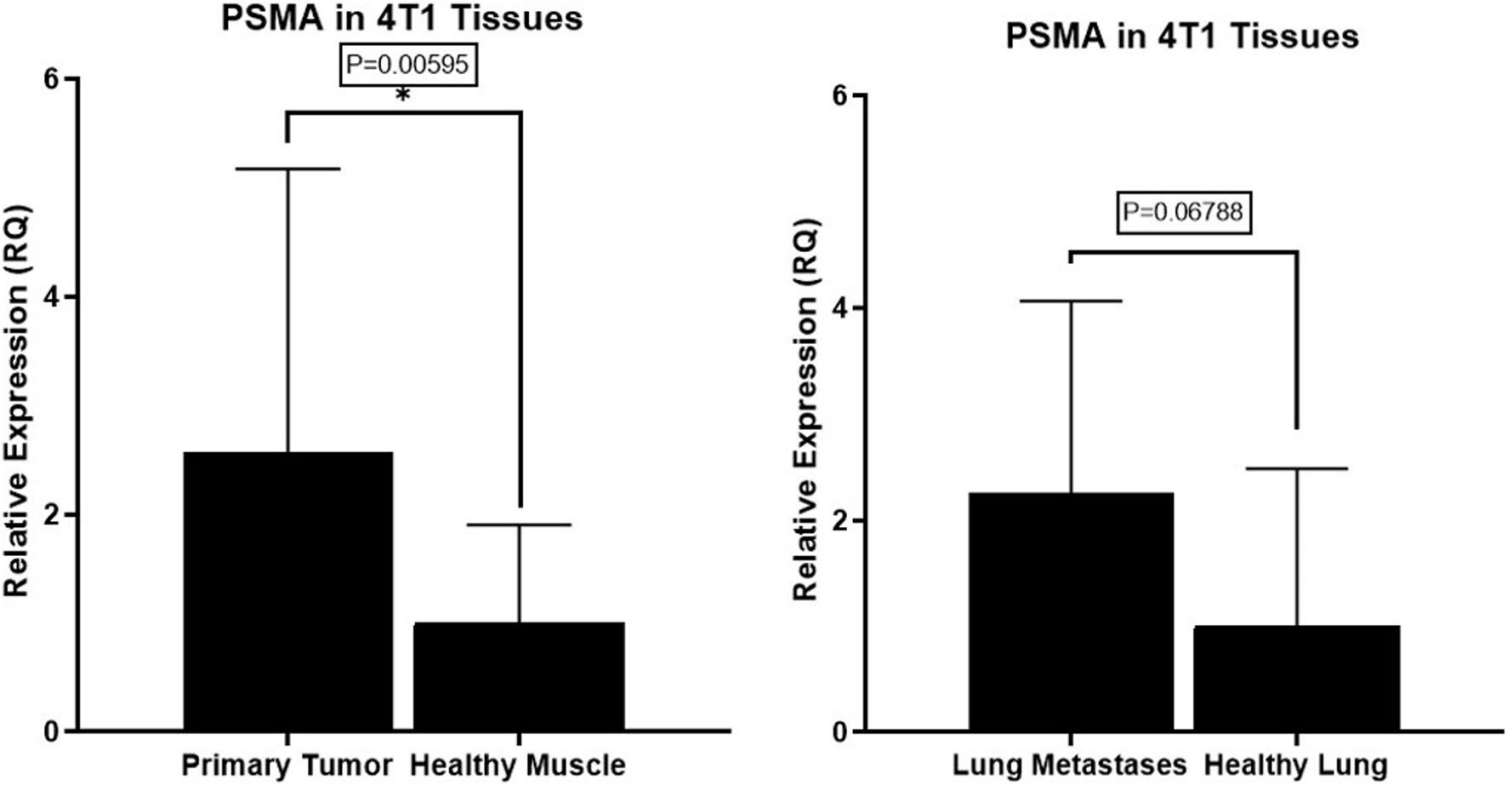
PSMA expression in BCa tumors confirmed through RT-PCR analysis of tissues. RT-PCR analysis of the same samples against mouse PSMA show significantly higher expression in primary tumor tissues compared to healthy muscle tissues ( * reaches statistical significance where p = 0.00595 < 0.05). Lung metastases also show increased PSMA expression, however, is non-significant compared to healthy lung tissues (p = 0.06788 > 0.05). The relative expression for primary tumors and tissues with lung metastases was compared against healthy muscle tissue and healthy lung tissue respectively. Values are mean ± SD of 4 mice

To compare the expression of PSMA in primary tumors and metastases, versus normal tissues we performed RT-PCR. Through analyzing murine 4T1 primary tumors as well as lung metastases from immunocompetent BALB/c and comparing to healthy lungs and muscle tissues using RT-PCR, significantly higher PSMA expression is observed when compared to non-cancerous tissues Fig. 5.

### PSMA Targeted Fluorescence Probes Can be Used to Visualize 4T1 Tumors *In Vivo*

**Fig. 6:**
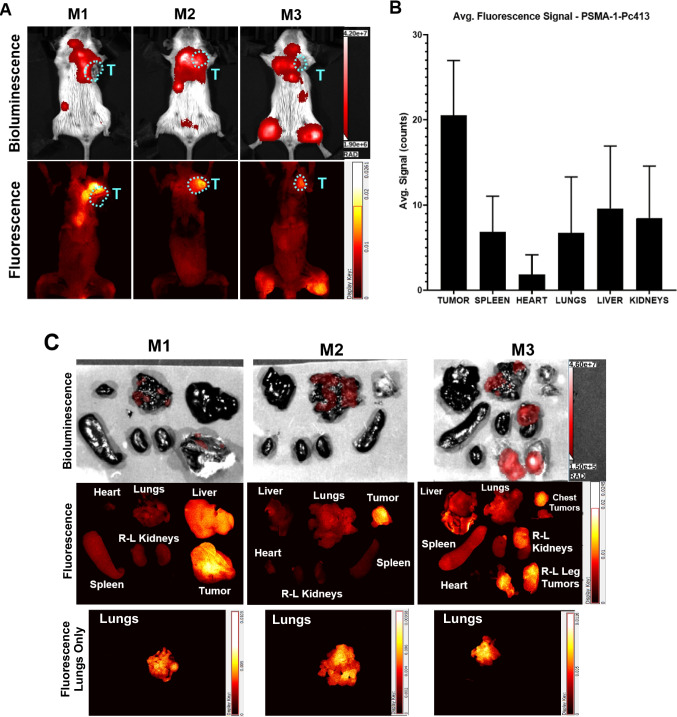
PSMA can be used as a target in mice with 4T1 tumors *In Vivo*. **(A)** Bioluminescence (BLI) & fluorescence imaging of BALB/c mice bearing an orthotopic 4T1-Luc with tumor highlighted by the cyan dashed circles denoted “T”. Images taken 24 h post-injection with 0.5 mg/kg PSMA-1-Pc413 showing accumulation of probe within the tumor and local and distant metastases. **(B)** Quantification of average pixel intensity over the area of the tissues, normalized to background, in the mice with primary and metastatic tumor tissues presents significantly higher signal than other tissues. The graph shows average signals 24 h after PSMA-1-Pc413 injection from 5 mice, images from 2 mice are presented in Fig. S11, fluorescence image of the organs as well as metastatic lungs on their own from M1-M3. We see a decay post-mortem compared to the signals in Fig. 6A. However, we can see all primary tumors present significant uptake as well as extensive distal metastases in M3 in the Kidney and hind limb tumors. **(C)** We can additionally observe PSMA-1-Pc413 uptake in the lung metastases (bottom row), although these signals are lower when compared to primary tumors and other more established metastatic masses. TBR Values: M1 Tumor 2.98; M2 Tumor 5.64; M3 Chest Tumors 1.62; M3 Kidneys 1.17; M3 Leg Tumors 2.21

To test if PSMA-targeted fluorescence probe can be accumulated by PSMA-associated tumor vasculature in 4T1 tumors to an extent for non-invasive fluorescence imaging, mice bearing orthotopic 4T1-Luc tumors were injected with PSMA-1-Pc413, and at 24 h both fluorescence and bioluminescence images were taken Fig. 6(A). Fluorescence imaging of these animals showed positive tumor accumulation of PSMA-1-Pc413 in the primary tumors on the top right mammary fat pad of the animals as well as lung metastases (chest) and distant lower leg metastases. Of these 5 animals, 3 developed extensive metastases in the lungs which were detectable through imaging using PSMA-1-Pc413. All tumors were confirmed through fluorescence imaging and bioluminescence imaging (BLI) indicating good uptake of the agent in these tissues. Additionally, one mouse (M3) displayed extensive metastases in the abdomen infecting the kidneys and liver which can also be visualized in the fluorescence images. A TBR > 1.5 is considered good visual contrast which allows physicians to differentiate between cancerous tissues and normal structures for identification and resection purposes [54, 55]. TBR values were greater than 1.5 for primary tumors in the mammary fat pad and leg metastases in M3 which means these tissues would be positively identified as cancerous through imaging with PSMA-1-Pc413. On extracting and imaging tissues, Fig. 6(C), significant uptake in the primary tumors and distant leg metastases was observed. The imaging agent was also able to selectively visualize lung metastases as can be seen in the fluorescence images of the lungs with metastases alone Fig. 6(C), (Suppl. Fig. S13, see ESM). Quantification of the fluorescence signal within the tissues Fig. 6(B) showed the most accumulation in the primary tumor and lung metastases, with minimal signal from the other organs. As can be seen from both fluorescence and bioluminescence imaging, advanced metastases were also seen on the kidneys and liver (right mouse). (see ESM). Plotting TBR values from this set of mice, (Suppl. Fig. S14B, see ESM**)** we see a gradual increase in TBR values starting from the 2-h timepoint. The TBR values are greater than 1.5 between 2—4 h, and therefore cancerous tissues can be positively identified using PSMA-1-Pc413 in this model. We see a significant increase in TBR between 5 min and 24 h (Paired t-test, p = 0.0181), as can be visualized through brighter tumor tissue in the representative mouse. While there is a slight increase between 24 and 72 h, it is not significant (Paired t-test p = 0.394), however, this is mainly due to great variation in TBR caused by minimal uptake in the primary tumor of M5 as compared to the other mice. Additionally, we quantified the fluorescence signal (Suppl. Fig. S14(A), see ESM) within the tumor along a time course and observed an increase in signal to a peak at 24 h (Suppl. Fig. S14(C), see ESM). This data suggests that PSMA-1-Pc413 can be used positively to identify 4T1 tumors.

Since BLI using Luciferin-Luciferase interaction depends on active metabolism, which is adenosine triphosphate (ATP) dependent, we can see BLI favors metastases within the lungs and distal metastases in the kidneys and limbs compared to the primary tumor. In contrast, PSMA-1-Pc413 shows the highest accumulation in larger more established tumors, which may be an effect of better vascular targeting. Additionally, on larger tumors as in (Suppl. Fig. S4, see ESM), we see loss of BLI signal on the primary tumors with tumors that have developed a scab, which presents a physical barrier to imaging, and may also possess a necrotic core where metabolic activity may be lower.

Analyzing PSMA-1-Pc413 in 4T1 tumors compared to athymic mice with high PSMA expressing PC3-Pip tumors, we can see (Suppl. Fig. S15, see ESM) that accumulation in 4T1 tumors is within the range of accumulation compared to PC3-Pip tumors. This suggests 4T1 tumors would be a good candidate for live fluorescence imaging.

## Discussion

FDA approved PSMA targeted imaging agents such as gallium(68)N-[N-[(S)-1,3-dicarboxypropyl] carbamoyl]-4-[(11)C]methyl-L-tyrosine (68 Ga-PSMA-11) and fluorine(18)N-[N-[(S)-1,3-dicarboxypropyl]carbamoyl]-4-[(18)F]methyl-L-tyrosine (piflufolastat F-18) are being used in clinical applications for the imaging of PCa [56, 57] and their application with PET/CT imaging for visualizing primary tumors and metastatic tissues for multiple solid human tumors, including BCa, in human patients has been confirmed through fluorine (18) 2-deoxy-2- [18F]fluoro-D-glucose (18F-FDG-PET/CT) as well as histological analysis [58–65]. With these PSMA targeted technologies, metastases were able to be visualized in the lungs, lymph nodes, and brain, and has shown comparable contrast to the primary tissues. Further, PSMA targeted theranostic agents and therapeutics for BCa, for example, a study by Von Hoff et al. using Docetaxel PSMA-targeted nanoparticles, have also been used to improve efficacy for the treatment of BCa in humans [66]. Our studies presented throughout this text have all shown links between PSMA and BCa and have shown evidence for successful PSMA targeting used in a non-prostatic cancer model. These studies highlight the many directions research could explore related to PSMA in BCa tissues. While these are successful examples, there is a lack of a mouse model to begin to explore different avenues for targeting the PSMA in tumors other than prostate tumor.

In our studies, we observed PSMA expression with the TNBC 4T1 tumor samples showing a significant overexpression compared to healthy tissues. Additionally, positive PSMA expression has been characterized to be colocalized on the tumor neovasculature in both tumors and lung metastases. The expression of PSMA in syngeneic breast tumors leads to significant tumor and metastases uptake when imaging with our PSMA-1 probes, highlighting its potential to be used as a biomolecular target. Our work here has begun to explore the expression of PSMA in the neovascular of mouse models of breast cancer and can enable other use of these models to study PSMA targeted agents for image guided surgery, therapeutics, radiosensitizers and so on in non-prostate cancers in animal models. Compared to other angiogenic targets such as vascular endothelial growth factor (VEGF) and integrins, that are also involved in the general process of angiogenesis, the tumor neovascular specificity of PSMA and the potential for internalization of PSMA makes it an ideal target, reducing off-target effects, and supports development of PSMA and vascular-targeted therapies and imaging techniques for BCa [67].

Heesch et al*.* [32] demonstrated PSMA expression in breast cancer stem cells (BCSCs) derived spheroids with a Human Vascular Endothelial Cell (HUVEC) co-culture *in vitro* system. Various human BCa cell lines, including MDA-MB-231 and MDA-MB-468, were created as HUVEC models and PSMA-CD31 colocalization in histological analysis images was observed. While these models validated PSMA as a target in human TNBC *in vitro* under diverse conditions, our studies confirmed PSMA-CD31 colocalization in histological analysis of human breast tissue samples *in vivo*.

Several histological and morphological studies examining angiogenesis and tumor vasculature in solid human cancers reveal irregular and diverse lumen structures, particularly in metastases [68, 69]. Understanding these structures is important as targeting strategies vary depending on neovascular expression. Research, for example Wang et al., has specifically investigated neovasculature in human breast cancer tissues to assess the progression and development of blood vessels in cancer [70].

The unique staining seen in the data presented here may be an artifact through immunofluorescence staining due to the abnormal structure of cancer vasculature which has been confirmed in our own study through H&E stains. Despite some tumor associated vasculature presenting as abnormal structures in metastases and primary syngeneic BCa tissues, we still observe PSMA expression further indicating that PSMA is a good vascular target in these tissues. The patterns and implications of PSMA expression within tumor neovasculature in non-prostatic cancers remain unclear. Our studies identify positive PSMA-CD31 expression; however, the overlap is heterogenous and varied in both primary tumor tissues and lung metastases. Additionally, while certain neovasculature present with PSMA expression, the overlap with CD31 remains minimal due signifying that these biomarkers occupy different areas morphologically. While this may mean that a portion of these tissues remain untargeted directly by PSMA, the irregular “leaky” structures of tumor vasculatures may allow these probes or therapies to infiltrate through the core of the tumor to areas that may show reduced PSMA expression. Additionally, therapies may further disrupt tumor structure allowing further penetration of targeted probes and drugs.

Although the novel use of PSMA as a biomarker in non-prostatic cancer in human patients has been studied previously, the applications of PSMA as a vascular targeted agent in these murine breast cancer models requires further research [32, 33]. This gap in research into FOLH1/PSMA expression is even more pronounced in murine cancer models. The purpose of this study is to characterize PSMA expression in murine TNBC 4T1 BCa tissues and therefore provide information for the development of PSMA-targeted diagnosis, treatment, and prognosis techniques. While this study shows positive correlation between PSMA-CD31 expression, tumor neovasculature highly varies from healthy vasculature in growth patterns and structures and so further analyses is required on the mechanism and significance of these results.

## Conclusion

We observed targetable levels of PSMA expression in the syngeneic, immunocompetent mouse models. There is an unmet need to improve BCa imaging and therapeutic systems to reduce repeat surgeries and treatments and potentially reduce harsh adjuvant therapies that patients undertake. Using these models, we can use varied PSMA-targeted therapies and imaging methods to develop and establish new alternative methods to improve BCa surgical and therapeutic treatments. PSMA holds great promise as an oncogenic target for BCa and its associated metastases. With these murine models, we can also look at previously approved or researched PSMA targeted therapies and imaging methods in clinical trials for prostate cancer and investigate their applications in BCa as with ^68^ Ga-PSMA studies. The implementation of these techniques in immunocompetent mice will also improve investigations of the immune response of these animals to various treatments in comparison to implanting human BCa cell lines in immunocompromised mice.

### Supplementary Information

Below is the link to the electronic supplementary material.

Supplementary file1 (DOCX 24 KB)

Supplementary file2 (DOCX 15 KB)

Supplementary file3 (DOCX 15 KB)

Supplementary file4 (PDF 2297 KB)

## Abbreviations

ATP: Adenosine triphosphate
BCa: Breast cancer
BCS: Breast conserving surgery
BLI: Bioluminescence imaging
cDNA: Complementary DNA
DMEM: Dulbecco’s modified eagle medium
ER: Estrogen receptor
FBS: Fetal bovine serum
FDA: United States Food and Drug Administration
FIGS: Fluorescence image guided surgery
FOLH1: Folate hydrolase – 1
GTEx: Genotype-tissue expression
H&E: Hematoxylin and eosin staining
HER2: Human endothelial growth factor 2
HUVEC: Human vascular endothelial cell
IC: Immunocompetent mice
IHC: Immunohistochemistry
IV: Intravenous
LNCaP: Lymph node carcinoma of the prostate cells
MC: Manders coefficient
Nu: Immunocompromised /athymic/ nude mice
OCT: Optimal cutting temperature
PBS: Phosphate-buffered saline
PCa: Prostate cancer
PCC: Pearsons correlation coefficient
PDT: Photodynamic therapy
PR: Progesterone receptor
PS: Penicillin-streptomycin solution
PSMA: Prostate specific membrane antigen
RNA-SEQ: Ribonucleic acid sequencing
RT-PCR: Reverse transcriptase – polymerase chain reaction
ROI: Region of interest
TCGA: The Cancer Genome Atlas
TIME: Tumor immune microenvironment
TNBC: Triple negative breast cancer
UCSC: University of California Santa Cruz
VEGF: Vascular endothelial growth factor
