# Supplementary material for: Prostate Specific Membrane Antigen Expression in a Syngeneic Breast Cancer Mouse Model
Source: Mol Imaging Biol. 2024 May 17;26(4):714–28. doi: 10.1007/s11307-024-01920-2 (PMC11281974; doi:10.1007/s11307-024-01920-2)
Supplement: Supplementary file 1 — Supplementary file1 (DOCX 24 KB) [file 11307_2024_1920_MOESM1_ESM.docx]

**Electronic Supplementary Material**

**Title:** Prostate Specific Membrane Antigen Expression in Syngeneic Breast Cancer Mouse Models

**Journal:** Molecular Imaging and Biology

**Authors:** **Aditi A. Shirke^1^**^*^, Jing Wang^2^, Gopolakrishnan Ramamurthy^2^, Arpan Mahanty^2^, Ethan Walker^1^, Lifang Zhang^2^, Xinning Wang^1*^, James P. Basilion ^1,2*^

^1^ Department of Biomedical Engineering, Case Western Reserve University, 11100 Euclid Ave, Wearn Building B-49, Cleveland, OH 44106, USA.

^2^ Department of Radiology, Case Western Reserve University, 11100 Euclid Ave, Wearn Building B-49, Cleveland, OH 44106, USA.

**^*^ Corresponding authors:**

**Xinning Wang**, Department of Biomedical Engineering, Case Western Reserve University, 11100 Euclid Ave, Wearn Building B-49, Cleveland, OH 44106, USA; **Tel:** +1-216-844-4848; **Email:** xxw171@case.edu

**James P. Basilion**, Department of Biomedical Engineering, Department of Radiology, Case Western Reserve University, 11100 Euclid Ave, Wearn Building B-49, Cleveland, OH 44106, USA; **Tel:** +1-216-502-1803; **Email:** jxb206@case.edu

**Shortened Title:** PSMA Expression in Syngeneic Breast Cancer Models

**Table.S1:** **UCSC Cohorts for Data Presented (obtained from the UCSC Xena Portal (https://xena.ucsc.edu/))**

| **Sample Count by Cohort** | | | | | | | | | | | | | | | |
| --- | --- | --- | --- | --- | --- | --- | --- | --- | --- | --- | --- | --- | --- | --- | --- |
| **Cancer Type** | **Total Count** | **Pathologic M** | | **Pathologic N** | | **Pathologic T** | | | | | | **Pathologic Stage** | | | |
|  |  | **M-** | **M+** | **N-** | **N+** | **Total** | **T0** | **T1** | **T2** | **T3** | **T4** | **I** | **II** | **III** | **IV** |
| ACC | 78 |  |  | 67 | 9 | 76 |  | 8 | 42 | 8 | 18 | 8 | 37 | 16 | 15 |
| DLBC | 46 |  |  |  |  |  |  |  |  |  |  |  |  |  |  |
| BLCA | 407 | 196 | 207 | 236 | 165 | 374 | 1 | 3 | 119 | 193 | 58 | 2 | 130 | 139 | 133 |
| BRCA | 1097 | 911 | 184 | 516 | 579 | 1092 |  | 280 | 634 | 138 | 40 | 182 | 621 | 250 | 20 |
| CESC | 302 | 115 | 137 | 130 | 122 | 239 |  | 140 | 70 | 20 | 9 |  |  |  |  |
| CHOL | 36 | 28 | 8 | 26 | 10 | 36 |  | 19 | 12 | 5 |  | 19 | 9 | 1 | 7 |
| COAD | 275 | 190 | 85 | 162 | 113 | 275 |  | 6 | 43 | 188 | 38 | 45 | 109 | 78 | 39 |
| ESCA | 184 | 135 | 27 | 76 | 91 | 167 | 1 | 31 | 42 | 88 | 5 | 19 | 78 | 56 | 9 |
| GBM | 154 |  |  |  |  |  |  |  |  |  |  |  |  |  |  |
| HNSC | 519 | 186 | 63 | 175 | 319 | 457 | 1 | 48 | 136 | 98 | 174 | 27 | 74 | 80 | 266 |
| KICH | 66 | 34 | 11 | 40 | 26 | 66 |  | 21 | 25 | 18 | 2 | 21 | 25 | 14 | 6 |
| KIRP | 289 | 95 | 179 | 50 | 238 | 287 |  | 192 | 33 | 60 | 2 | 171 | 21 | 52 | 15 |
| LGG | 515 |  |  |  |  |  |  |  |  |  |  |  |  |  |  |
| LIHC | 371 | 266 | 105 | 252 | 118 | 368 |  | 181 | 94 | 80 | 13 | 171 | 86 | 85 | 5 |
| LUAD | 514 | 346 | 164 | 330 | 183 | 511 |  | 169 | 276 | 47 | 19 | 274 | 122 | 84 | 26 |
| LUSC | 502 | 411 | 86 | 319 | 182 | 501 |  | 114 | 293 | 71 | 23 | 244 | 162 | 84 | 7 |
| LUNG | 1017 | 757 | 251 | 650 | 365 | 1013 |  | 283 | 570 | 118 | 42 | 519 | 284 | 168 | 33 |
| MESO | 87 | 57 | 30 | 44 | 43 | 85 |  | 14 | 26 | 32 | 13 | 10 | 16 | 45 | 16 |
| OV | 303 |  |  |  |  |  |  |  |  |  |  |  |  |  |  |
| PAAD | 178 | 80 | 98 | 48 | 128 | 176 |  | 7 | 24 | 142 | 3 | 21 | 147 | 4 | 4 |
| PCPG | 179 |  |  |  |  |  |  |  |  |  |  |  |  |  |  |
| PRAD | 497 |  |  | 345 | 79 | 490 |  |  | 187 | 293 | 10 |  |  |  |  |
| READ | 94 | 65 | 26 | 40 | 52 | 92 |  | 4 | 13 | 65 | 10 | 12 | 26 | 33 | 13 |
| SARC | 257 |  |  |  |  |  |  |  |  |  |  |  |  |  |  |
| SKCM | 103 | 98 | 3 | 58 | 44 | 101 |  | 1 | 5 | 10 | 85 | 2 | 66 | 27 | 3 |
| STAD | 414 | 366 | 48 | 123 | 290 | 405 |  | 22 | 88 | 180 | 115 | 57 | 123 | 170 | 41 |
| TGCT | 150 | 115 | 4 | 46 | 78 | 134 |  | 76 | 51 | 6 | 1 | 55 | 12 | 14 |  |
| THCA | 505 | 282 | 222 | 230 | 275 | 503 |  | 143 | 166 | 171 | 23 | 284 | 52 | 112 | 55 |
| THYM | 113 |  |  |  |  |  |  |  |  |  |  |  |  |  |  |
| UCEC | 176 |  |  |  |  |  |  |  |  |  |  |  |  |  |  |
| UCS | 57 |  |  |  |  |  |  |  |  |  |  |  |  |  |  |
| UVM | 80 | 51 | 27 | 52 | 27 | 80 |  |  | 14 | 32 | 34 |  | 39 | 36 | 4 |

**ACC**: Adrenocortical Carcinoma, **DLBC**: Lymphoid Neoplasm Diffuse Large B-cell Lymphoma, **BLCA**: Bladder Urothelial Carcinoma, **BRCA**: Breast invasive carcinoma, **CESC**: Cervical squamous cell carcinoma and endocervical adenocarcinoma, **CHOL**: Cholangiocarcinoma, **COAD**: Colon adenocarcinoma, **ESCA**; Esophageal carcinoma, **GBM**: Glioblastoma multiforme, **HNSC**: Head and Neck squamous cell carcinoma, **KICH**: Kidney Chromophobe, KIRP: Kidney renal papillary cell carcinoma, **LGG**: Brain Lower Grade Glioma, **LIHC**: Liver hepatocellular carcinoma, **LUAD**: Lung adenocarcinoma, **LUSC**: Lung squamous cell carcinoma, **LUNG**: Lung cancer, **MESO**: Mesothelioma, **OV**: Ovarian serous cystadenocarcinoma, **PAAD**: Pancreatic adenocarcinoma, **PCPG**: Pheochromocytoma and Paraganglioma, **PRAD**: Prostate adenocarcinoma, **READ**: Rectum adenocarcinoma, **SARC**: Sarcoma, **SKCM**: Skin Cutaneous Melanoma, **STAD**: Stomach adenocarcinoma, **TGCT**: Testicular Germ Cell Tumors, **THCA**: Thyroid carcinoma, **THYM**: Thymoma, **UCEC**: Uterine Corpus Endometrial Carcinoma, **UCS**: Uterine Carcinosarcoma, **UVM**: Uveal Melanoma
