# Supplementary material for: Prostate Specific Membrane Antigen Expression in a Syngeneic Breast Cancer Mouse Model
Source: Mol Imaging Biol. 2024 May 17;26(4):714–28. doi: 10.1007/s11307-024-01920-2 (PMC11281974; doi:10.1007/s11307-024-01920-2)
Supplement: Supplementary file 2 — Supplementary file2 (DOCX 15 KB) [file 11307_2024_1920_MOESM2_ESM.docx]

# **Table.S2: List of all Reagents Used for Immunofluorescence Staining**

| **Reagent Name** | **Molecular Target** | **Ab Source** | **Reactivity** | **Vendor Details** |
| --- | --- | --- | --- | --- |
| PSMA-1-Alexa488 | PSMA | n/a | Mouse + Human cell lines | Developed and synthesized in Basilion Lab |
| Anti-PSMA | PSMA | Rabbit IgG - Monoclonal | Mouse + Human cell lines | Cell Signaling Technology - Prostate Specific Membrane Antigen (D7I8E) XP® Rabbit mAb #12815 |
| Anti-CD31 | CD31 | Rabbit IgG - Polyclonal | Mouse + Human cell lines | Abcam - Anti-CD31 antibody (ab28364) |
| Hoechst Solution | DNA - Nucleus |  |  | Thermo Fisher - Hoechst 33342, Trihydrochloride, Trihydrate - 10 mg/mL Solution in Water |
| Secondary Antibody | | | | |
| **Reagent Name** | **Species Reactivity** | | **Host/Isotype** | **Vendor Details** |
| Alexa Fluor™ 594 | Rabbit | | Goat/IgG | Thermo Fisher - Goat anti-Rabbit IgG (H+L) Cross-Adsorbed ReadyProbes™ Secondary Antibody, Alexa Fluor™ 594 |
| Alexa Fluor™ 488 | Rabbit | | Goat/IgG | Thermo Fisher - Goat anti-Rabbit IgG (H+L) Cross-Adsorbed Secondary Antibody, Alexa Fluor™ 488 |
