# Supplementary material for: Prostate Specific Membrane Antigen Expression in a Syngeneic Breast Cancer Mouse Model
Source: Mol Imaging Biol. 2024 May 17;26(4):714–28. doi: 10.1007/s11307-024-01920-2 (PMC11281974; doi:10.1007/s11307-024-01920-2)
Supplement: Supplementary file 3 — Supplementary file3 (DOCX 15 KB) [file 11307_2024_1920_MOESM3_ESM.docx]

# **Table.S3: PCC and MC values**

| **Figure Number** | **Position** | **PCC** | **MC** |
| --- | --- | --- | --- |
| Fig.1B | Top row - MDA-MB-231 | 0.147 | 0.071 |
| Fig.1B | Bottom row - MDA-MB-468 | 0.316 | 0.607 |
| Fig.2A | Column 1-Primary Tumor-IC | 0.534 | 0.381 |
| Fig.2A | Column 2-Primay Tumor-Nu | 0.627 | 0.541 |
| Fig.2A | Column 3-Infiltrative tumor -IC | 0.336 | 0.281 |
| Fig.3 | Primary Tumor | 0.610* | 0.200** |
| Fig.3 | Lung Metastases | 0.617* | 0.240** |
| Fig.4 | Top row - Lung Metastases- immunocompetent mice | 0.711 | 0.801 |
| Fig.4 | Bottom row - Lung Metastases- Athymic/Nu BALB/c mice | 0.481 | 0.320 |
|  |  |  |  |
| Fig. S5 | B | 0.396 | 0.559 |
| Fig. S11 | Primary Tumor (+ve PSMA expression) | 0.513 | 0.576 |
| Fig. S11 | Primary Tumor (-ve PSMA expression) | 0.387 | 0.296 |
| Fig. S11 | Lung Metastases (+ve PSMA expression) | 0.747 | 0.636 |
| Fig. S11 | Lung Metastases (+ve PSMA expression) | 0.129 | 0.158 |

*average PCC value, calculated through a Fisher Transform Indicator.

**geometric mean MC values.
