# Supplementary material for: Prostate Specific Membrane Antigen Expression in a Syngeneic Breast Cancer Mouse Model
Source: Mol Imaging Biol. 2024 May 17;26(4):714–28. doi: 10.1007/s11307-024-01920-2 (PMC11281974; doi:10.1007/s11307-024-01920-2)
Supplement: Supplementary file 4 — Supplementary file4 (PDF 2297 KB) [file 11307_2024_1920_MOESM4_ESM.pdf]

**Electronic Supplementary Material**

**Title:** Prostate Specific Membrane Antigen Expression in a Syngeneic Breast Cancer Mouse Model

**Journal:** Molecular Imaging and Biology

**Authors: Aditi A. Shirke[1*]**, Jing Wang[2], Gopolakrishnan Ramamurthy[2], Arpan Mahanty[2], Ethan Walker[1], Lifang Zhang[2], Abhiram Panigrahi2, Xinning Wang[1*], James P. Basilion [1,2*]

[1] Department of Biomedical Engineering, Case Western Reserve University, 11100 Euclid Ave, Wearn Building B-49, Cleveland, OH 44106, USA.

[2] Department of Radiology, Case Western Reserve University, 11100 Euclid Ave, Wearn Building B-49, Cleveland, OH 44106, USA.

**[*] Corresponding authors:**

**Aditi A. Shirke**, Department of Biomedical Engineering, Case Western Reserve University, 11100 Euclid Ave, Wearn Building B-49, Cleveland, OH 44106, USA; **Tel:** +1-216-556-3950; **Email:** aas151@case.edu

**Xinning Wang**, Department of Biomedical Engineering, Case Western Reserve University, 11100 Euclid Ave, Wearn Building B-49, Cleveland, OH 44106, USA; **Tel:** +1-216-844-4848; **Email:** xxw171@case.edu

**James P. Basilion**, Department of Biomedical Engineering, Department of Radiology, Case Western Reserve University, 11100 Euclid Ave, Wearn Building B-49, Cleveland, OH 44106, USA; **Tel:** +1-216-502-1803; **Email:** jxb206@case.edu

**Shortened Title:** PSMA Expression in a Syngeneic Breast Cancer Model

**Table.S1: UCSC Cohorts for Data Presented (obtained from the UCSC Xena Portal (https://xena.ucsc.edu/))**

| Sample Count by Cohort | | | | | | | | | | | | | | | |
|---|---|---|---|---|---|---|---|---|---|---|---|---|---|---|---|
| **Cancer Type** | **Total Count** | **Pathologic M** | | **Pathologic N** | | **Pathologic T** | | | | | | **Pathologic Stage** | | | |
| | | **M-** | **M+** | **N-** | **N+** | **Total** | **T0** | **T1** | **T2** | **T3** | **T4** | **I** | **II** | **III** | **IV** |
| ACC | 78 | | | 67 | 9 | 76 | | 8 | 42 | 8 | 18 | 8 | 37 | 16 | 15 |
| DLBC | 46 | | | | | | | | | | | | | | |
| BLCA | 407 | 196 | 207 | 236 | 165 | 374 | 1 | 3 | 119 | 193 | 58 | 2 | 130 | 139 | 133 |
| BRCA | 1097 | 911 | 184 | 516 | 579 | 1092 | | 280 | 634 | 138 | 40 | 182 | 621 | 250 | 20 |
| CESC | 302 | 115 | 137 | 130 | 122 | 239 | | 140 | 70 | 20 | 9 | | | | |
| CHOL | 36 | 28 | 8 | 26 | 10 | 36 | | 19 | 12 | 5 | | 19 | 9 | 1 | 7 |
| COAD | 275 | 190 | 85 | 162 | 113 | 275 | | 6 | 43 | 188 | 38 | 45 | 109 | 78 | 39 |
| ESCA | 184 | 135 | 27 | 76 | 91 | 167 | 1 | 31 | 42 | 88 | 5 | 19 | 78 | 56 | 9 |
| GBM | 154 | | | | | | | | | | | | | | |
| HNSC | 519 | 186 | 63 | 175 | 319 | 457 | 1 | 48 | 136 | 98 | 174 | 27 | 74 | 80 | 266 |
| KICH | 66 | 34 | 11 | 40 | 26 | 66 | | 21 | 25 | 18 | 2 | 21 | 25 | 14 | 6 |
| KIRP | 289 | 95 | 179 | 50 | 238 | 287 | | 192 | 33 | 60 | 2 | 171 | 21 | 52 | 15 |
| LGG | 515 | | | | | | | | | | | | | | |

| | | | | | | | | | | | | | | | |
|---|---|---|---|---|---|---|---|---|---|---|---|---|---|---|---|
| LIHC | 371 | 266 | 105 | 252 | 118 | 368 | | 181 | 94 | 80 | 13 | 171 | 86 | 85 | 5 |
| LUAD | 514 | 346 | 164 | 330 | 183 | 511 | | 169 | 276 | 47 | 19 | 274 | 122 | 84 | 26 |
| LUSC | 502 | 411 | 86 | 319 | 182 | 501 | | 114 | 293 | 71 | 23 | 244 | 162 | 84 | 7 |
| LUNG | 1017 | 757 | 251 | 650 | 365 | 1013 | | 283 | 570 | 118 | 42 | 519 | 284 | 168 | 33 |
| MESO | 87 | 57 | 30 | 44 | 43 | 85 | | 14 | 26 | 32 | 13 | 10 | 16 | 45 | 16 |
| OV | 303 | | | | | | | | | | | | | | |
| PAAD | 178 | 80 | 98 | 48 | 128 | 176 | | 7 | 24 | 142 | 3 | 21 | 147 | 4 | 4 |
| PCPG | 179 | | | | | | | | | | | | | | |
| PRAD | 497 | | | 345 | 79 | 490 | | | 187 | 293 | 10 | | | | |
| READ | 94 | 65 | 26 | 40 | 52 | 92 | | 4 | 13 | 65 | 10 | 12 | 26 | 33 | 13 |
| SARC | 257 | | | | | | | | | | | | | | |
| SKCM | 103 | 98 | 3 | 58 | 44 | 101 | | 1 | 5 | 10 | 85 | 2 | 66 | 27 | 3 |
| STAD | 414 | 366 | 48 | 123 | 290 | 405 | | 22 | 88 | 180 | 115 | 57 | 123 | 170 | 41 |
| TGCT | 150 | 115 | 4 | 46 | 78 | 134 | | 76 | 51 | 6 | 1 | 55 | 12 | 14 | |
| THCA | 505 | 282 | 222 | 230 | 275 | 503 | | 143 | 166 | 171 | 23 | 284 | 52 | 112 | 55 |
| THYM | 113 | | | | | | | | | | | | | | |
| UCEC | 176 | | | | | | | | | | | | | | |
| UCS | 57 | | | | | | | | | | | | | | |
| UVM | 80 | 51 | 27 | 52 | 27 | 80 | | | 14 | 32 | 34 | | 39 | 36 | 4 |

**ACC**: Adrenocortical Carcinoma, **DLBC**: Lymphoid Neoplasm Diffuse Large B-cell Lymphoma, **BLCA**: Bladder Urothelial Carcinoma, **BRCA**: Breast invasive carcinoma, **CESC**: Cervical squamous cell carcinoma and endocervical adenocarcinoma, **CHOL**: Cholangiocarcinoma, **COAD**: Colon adenocarcinoma, **ESCA**; Esophageal carcinoma, **GBM**: Glioblastoma multiforme, **HNSC**: Head and Neck squamous cell carcinoma, **KICH**: Kidney Chromophobe, KIRP: Kidney renal papillary cell carcinoma, **LGG**: Brain Lower Grade Glioma, **LIHC**: Liver hepatocellular carcinoma, **LUAD**: Lung adenocarcinoma, **LUSC**: Lung squamous cell carcinoma, **LUNG**: Lung cancer, **MESO**: Mesothelioma, **OV**: Ovarian serous cystadenocarcinoma, **PAAD**: Pancreatic adenocarcinoma, **PCPG**: Pheochromocytoma and Paraganglioma, **PRAD**: Prostate adenocarcinoma, **READ**: Rectum adenocarcinoma, **SARC**: Sarcoma, **SKCM**: Skin Cutaneous Melanoma, **STAD**: Stomach adenocarcinoma, **TGCT**: Testicular Germ Cell Tumors, **THCA**: Thyroid carcinoma, **THYM**: Thymoma, **UCEC**: Uterine Corpus Endometrial Carcinoma, **UCS**: Uterine Carcinosarcoma, **UVM**: Uveal Melanoma

**Table.S2: List of all Reagents Used for Immunofluorescence Staining**

| Reagent Name | Molecular Target | Ab Source | Reactivity | Vendor Details |
|---|---|---|---|---|
| PSMA-1-Alexa488 | PSMA | N/A | Mouse + Human cell lines | Developed and synthesized in Basilion Lab |
| Anti-PSMA | PSMA | Rabbit IgG - Monoclonal | Mouse + Human cell lines | Cell Signaling Technology - Prostate Specific Membrane Antigen (D7I8E) XP® Rabbit mAb #12815 |
| Anti-CD31 | CD31 | Rabbit IgG - Polyclonal | Mouse + Human cell lines | Abcam - Anti-CD31 antibody (ab28364) |
| Hoechst Solution | DNA - Nucleus | | | Thermo Fisher - Hoechst 33342, Trihydrochloride, Trihydrate - 10 mg/mL Solution in Water |
| Secondary Antibody | | | | |
| **Reagent Name** | **Species Reactivity** | | **Host/Isotype** | **Vendor Details** |
| Alexa Fluor™ 594 | Rabbit | | Goat/IgG | Thermo Fisher - Goat anti-Rabbit IgG (H+L) Cross-Adsorbed ReadyProbes™ Secondary Antibody, Alexa Fluor™ 594 |
| Alexa Fluor™ 488 | Rabbit | | Goat/IgG | Thermo Fisher - Goat anti-Rabbit IgG (H+L) Cross-Adsorbed Secondary Antibody, Alexa Fluor™ 488 |

**Table.S3: PCC and MC values**

| Figure Number | Position | PCC | MC |
|---|---|---|---|
| Fig.1B | Top row - MDA-MB-231 | 0.147 | 0.071 |
| Fig.1B | Bottom row - MDA-MB-468 | 0.316 | 0.607 |
| Fig.2A | Column 1-Primary Tumor-IC | 0.534 | 0.381 |
| Fig.2A | Column 2-Primay Tumor-Nu | 0.627 | 0.541 |
| Fig.2A | Column 3-Infiltrative tumor -IC | 0.336 | 0.281 |
| Fig.3 | Primary Tumor | 0.610* | 0.200** |
| Fig.3 | Lung Metastases | 0.617* | 0.240** |
| Fig.4 | Top row - Lung Metastases- immunocompetent mice | 0.711 | 0.801 |
| Fig.4 | Bottom row - Lung Metastases- Athymic/Nu BALB/c mice | 0.481 | 0.320 |
| Electronic Supplementary Information | | | |
| Fig. S5 | B | 0.396 | 0.559 |
| Fig. S11 | Primary Tumor (+ve PSMA expression) | 0.513 | 0.576 |
| Fig. S11 | Primary Tumor (-ve PSMA expression) | 0.387 | 0.296 |
| Fig. S11 | Lung Metastases (+ve PSMA expression) | 0.747 | 0.636 |
| Fig. S11 | Lung Metastases (+ve PSMA expression) | 0.129 | 0.158 |

*average PCC value, calculated through a Fisher Transform Indicator.
**geometric mean MC values.

Please note that data between certain sets of data cannot be compared, however, all 4T1 images that have been processed for determining PCC and MC values can be compared due to identical imaging settings and conditions between the images.

**Fig.S1: Structure of PSMA-1 ligand and PSMA-1-Pc413**

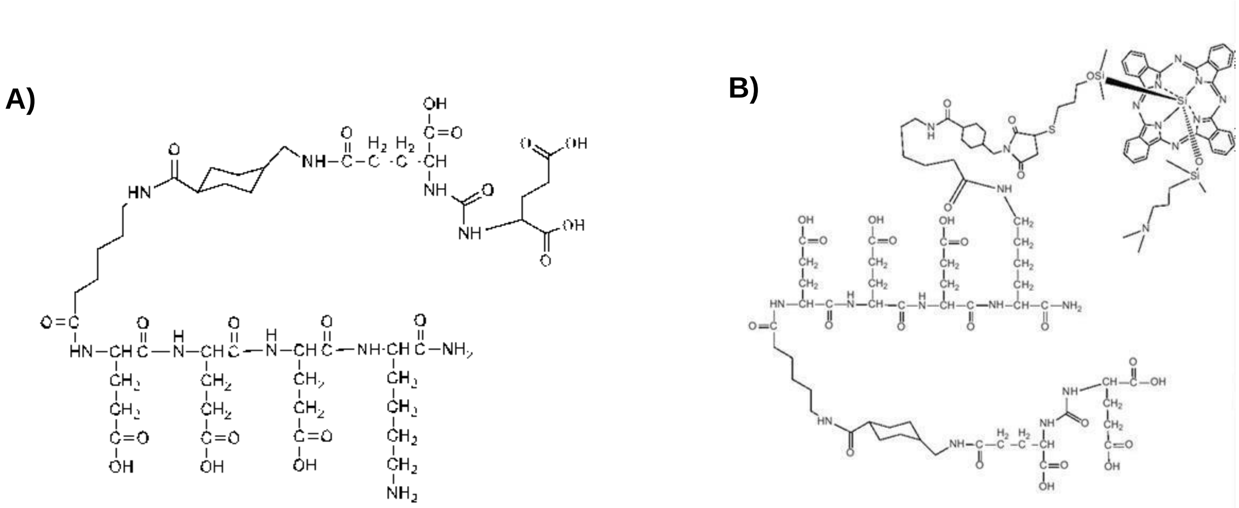


**Fig.S1:** Structure of our highly specific and selective PSMA-1 Ligand **(A)** which was used to create unique PSMA targeted imaging and treatment agents such as PSMA-1-Pc413 **(B)** used in *in vivo* models. We utilized PSMA-1-Pc413 to validate the accumulation of a PSMA-targeted probe by combining our PSMA-1 ligand with a phthalocyanine-based theranostic agent, Pc413. This compound effectively targets PSMA, allowing the visualization of prostate cancer tissues through high-fluorescence yield.

**Fig.S2: Synthesis and Characterization of PSMA-1-Alexa488**

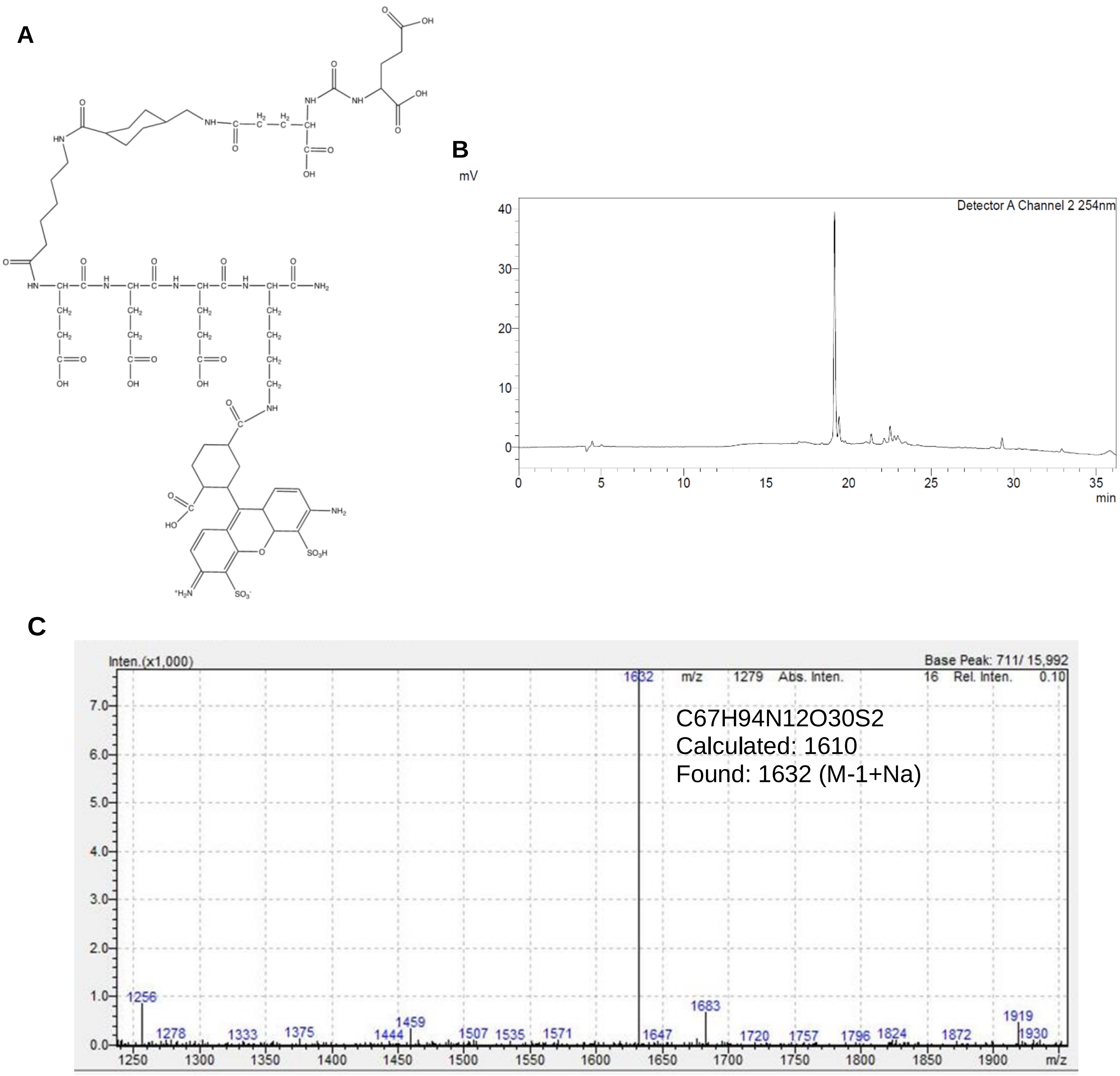


**Fig. S2: (A)** Chemical structure of PSMA-1-Alexa488 **(B)** HPLC chromatography output for the purification process of PSMA-1-Alexa488 with a retention time of 19.4 minutes. **(C)** MALDI-MS of PSMA-1-Alexa488 with the sharp peak at around 1632 signifying the structure.

**Fig.S3: Validation of PSMA-1-Alexa488 Fluorescence and PSMA Selectivity**

A

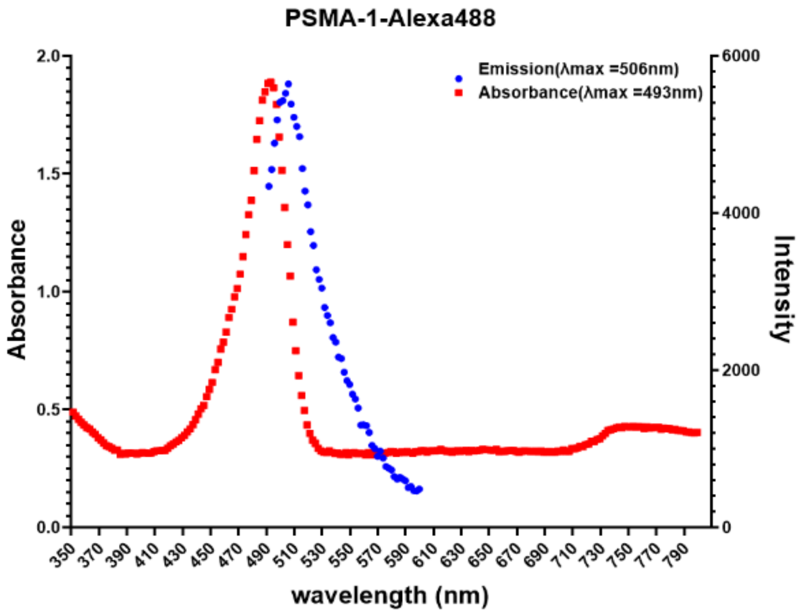


B

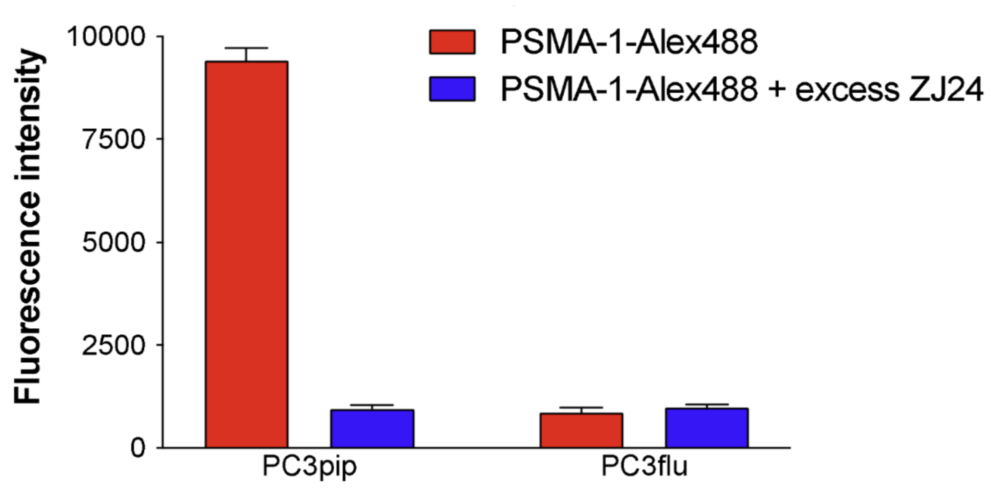


**Fig.S3: (A)** Overlay of Emission spectrum of diode laser (blue) and absorbance spectrum (red) of PSMA-1-Alexa488 (red). The maximum emission is 506nm and maximum absorbance is 493nm. **(B)** *In Vitro* study confirming specificity of PSMA-1-Alex488 to selectively target PSMA positive PC3-pip cells compared to PC3-flu cells. On addition of excess ZJ24 which block PSMA receptors we only observe non-specific uptake in both cell lines

**Fig.S4: Growth Rate of 4T1-Luc Primary Tumors and Metastases on Inoculation with Varying Amount of cells**

A) Bioluminescence Images of Female BALB/c Mice Inoculated with 4T1-Luc Cells

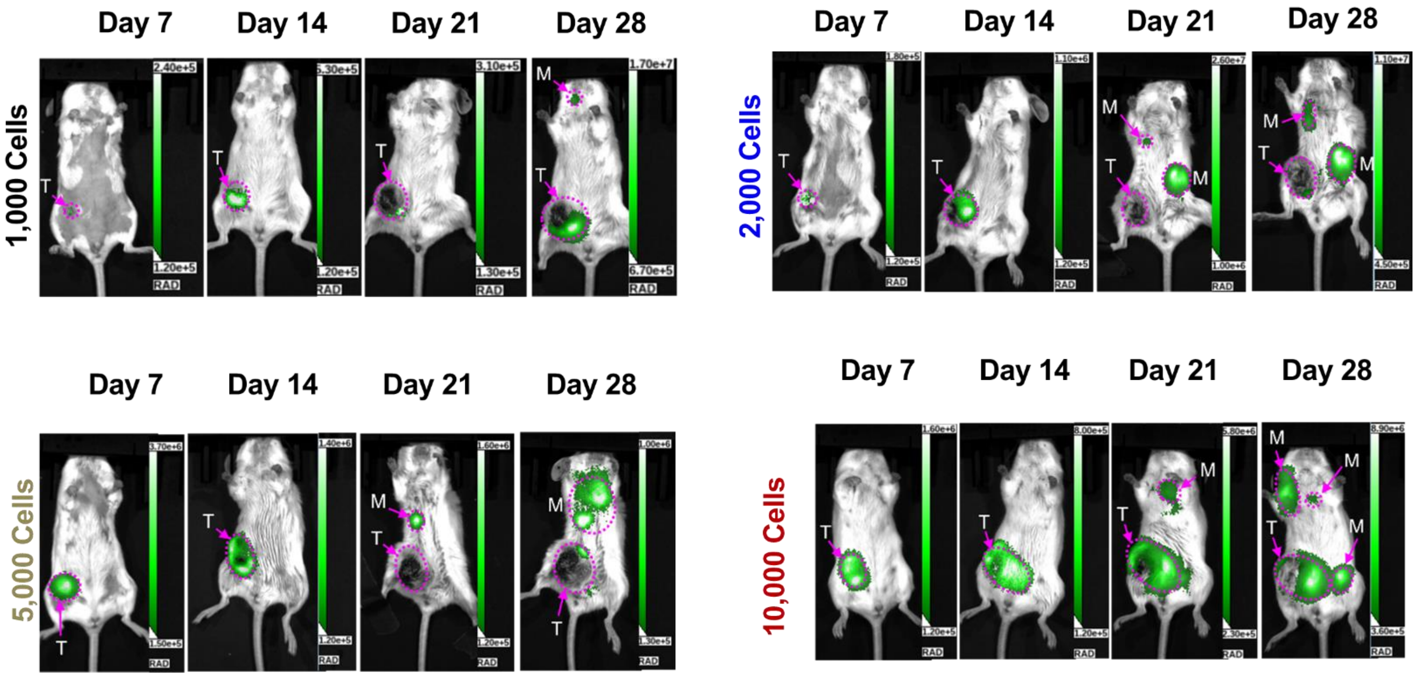


B)

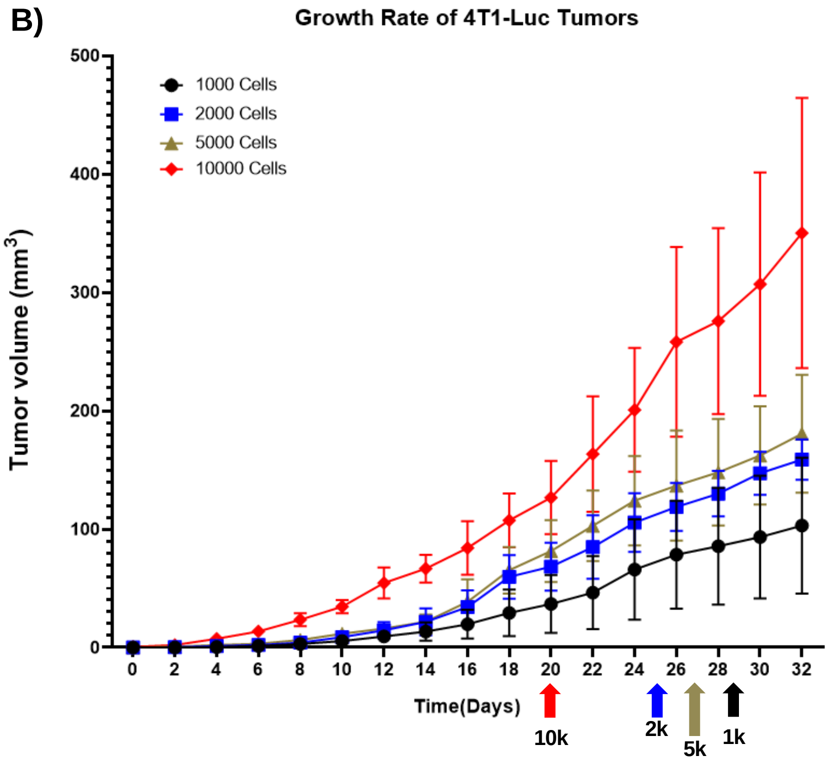

**Fig. S4: (A)** Female BALB/c mice were orthotopically inoculated with varying amounts of 4T1-Luc cells – 1,000 cells, 2,000 cells, 5,000 cells and 10,000 cells – with five mice per group. Primary tumor growth and metastases formation was tracked through Bioluminescence imaging. Four representative mice are shown here with weekly images till four weeks post-inoculation. Primary tumor locations are denoted with a **T** while metastases are denoted with a **M**. Please note, all images are on their own individual scale to highlight what was visualized on capturing the image **(B)** Primary tumor growth was measured through calipers every other day and plotted with the arrows under the grow designating the average day, between all 5 mice per group, at which metastases were detected through Bioluminescence imaging in the respective groups and all 25 mice showed metastases formation by Day 30. 10,000 cells naturally showed the highest growth rate and earliest metastases formation, appearing at around Days 18-22 after inoculation.

**Fig.S5: Process of Extracting Manders Coefficient and Pearsons Correlation Coefficient Values from Fluorescence Microscopy Images using JACoP**

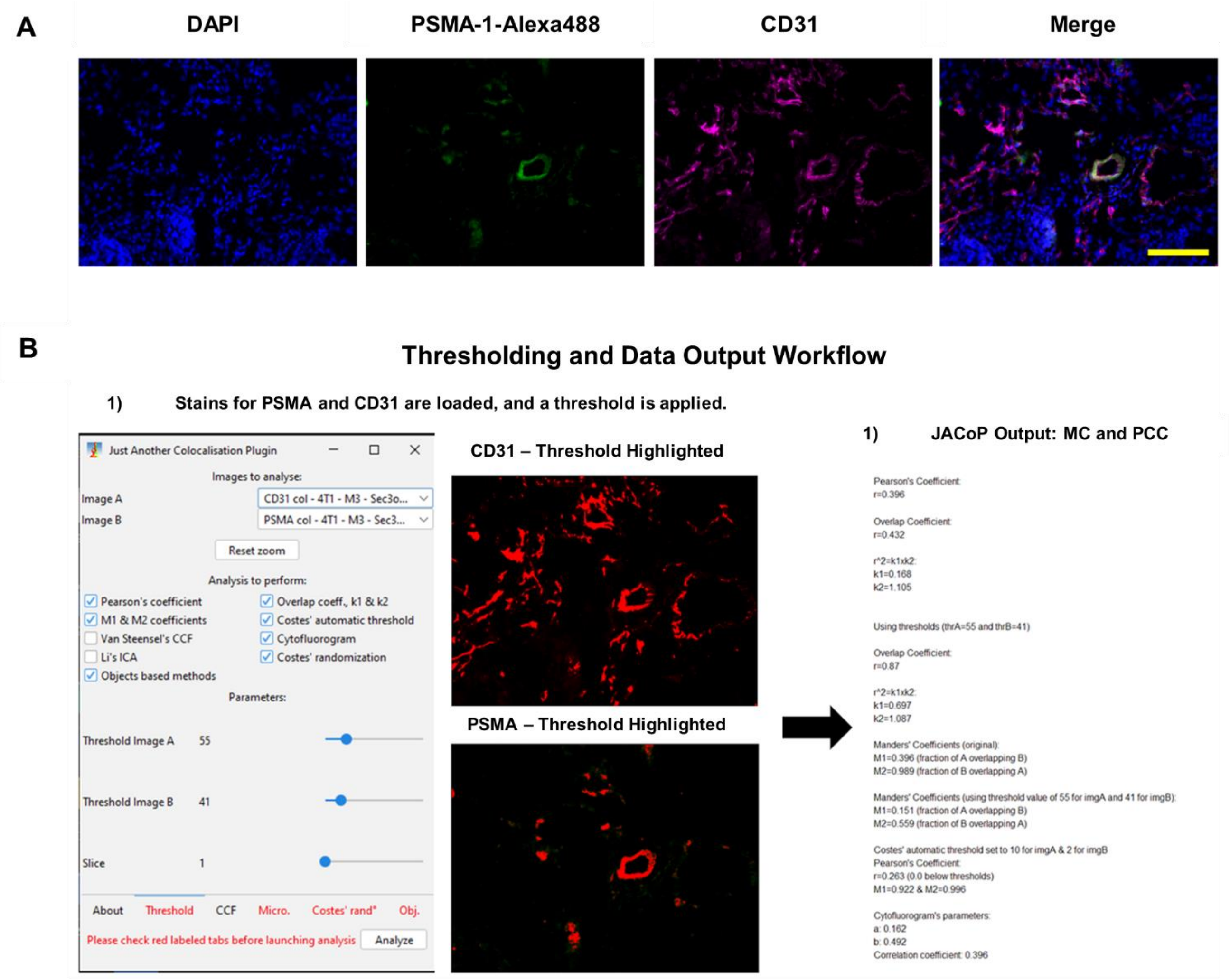


**Fig.S5: (A)** As with previous examples, these images show PSMA-CD31 staining and colocalization in a sample of 4T1 primary tumor tissue. Yellow bar measures 100 μm. **(B)** These images were then loaded into the JACoP plugin on Fiji and a threshold was applied as shown above. The red areas that are highlighted are considered "positive signal" and so using measurements such as pixel intensity and pixel position, the plugin outputs various selected measurements and metrics such as MC and PCC values. For this set of stains, MC = 0.559 and PCC = 0.396 at the thresholds visualized above.

**Fig.S6 - FOLH1 is Expressed in Multiple Solid Human Tumors**

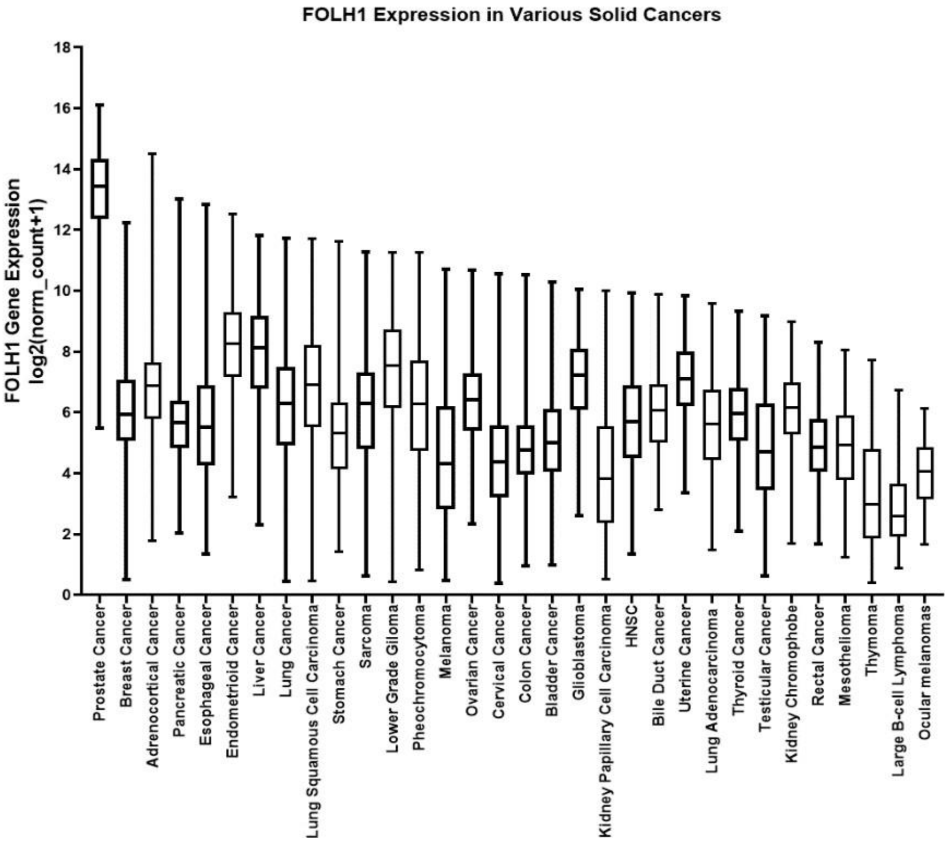


**Fig.S6:** PSMA, encoded by the FOLH1 gene, is expressed in prostate cancer as well as in many solid human cancers which leads to overexpression of PSMA transmembrane protein. FOLH1 expression in human samples from the UCSC Xena visual database which extracts data from the TCGA, GTex and multiple other publicly available cancer genomics and proteomics databases, shows FOLH1 expression levels within the prostate cancer levels. Plotting RNA-Seq data for FOLH1 gene expression extracted from this compilation of databases as displayed in **Table.S1**, we can observe that most solid human cancers show PSMA expression comparable to prostate cancer. Even utilizing databases like UCSC Xena, which compile data from various studies, there's limited information on FOLH1 gene and PSMA protein expression across different organ tissues—both malignant and benign. While the presented studies extensively analyze human samples and applications of PSMA-targeted

technologies, further research is needed to investigate FOLH1/PSMA expression and its clinical relevance in non-prostatic cancers using human models.

**Fig.S7: Quantification of Fluorescence from Human Cancer Tumors**

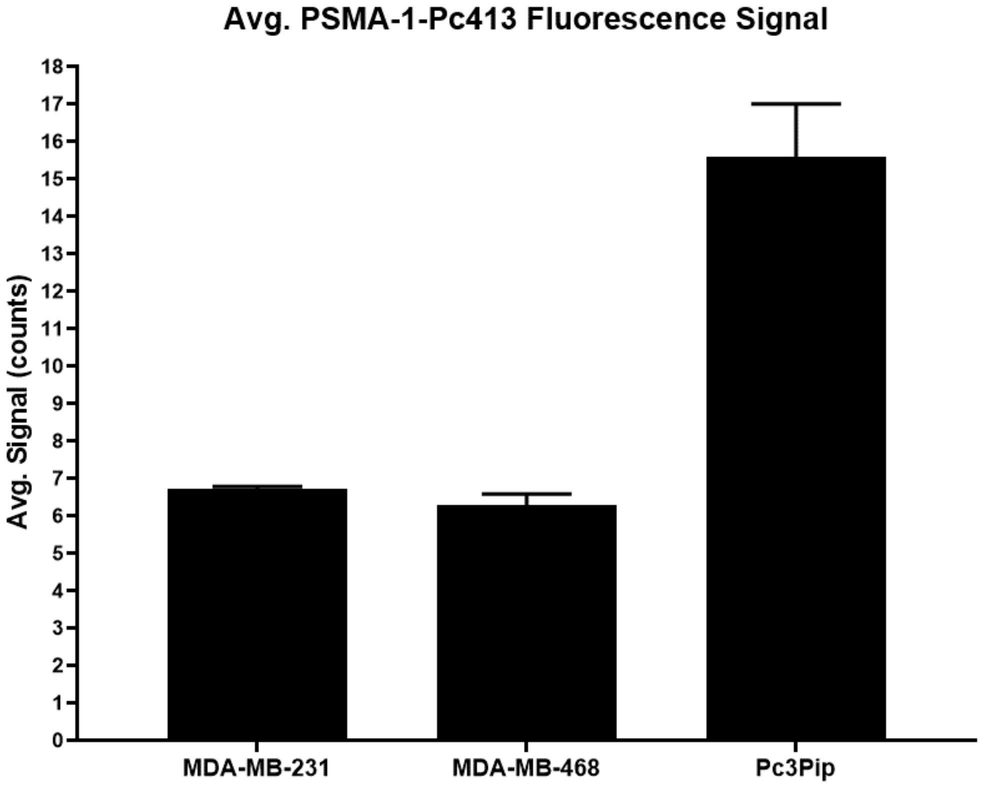


**Fig. S7:** Quantification of fluorescence signals 24 hours after IV injection of PSMA-1-Pc413 in athymic mice with orthotopic MDA-MB-231, MDA-MB-468 tumors, from **Fig. 1** in the main text, and flank PC3-Pip Tumors. MDA-MB-231 and MDA-MB-468 shows roughly equal accumulation of PSMA-1-Pc413. While accumulation is lower in BCa tissues than in Pc3-Pip tumors, the tumors still show good contrast during whole body imaging of these mice.

**Fig.S8: Additional PSMA-CD31 Staining in 4T1 Tumors to Show PSMA Colocalization on Tumor Neovasculature**

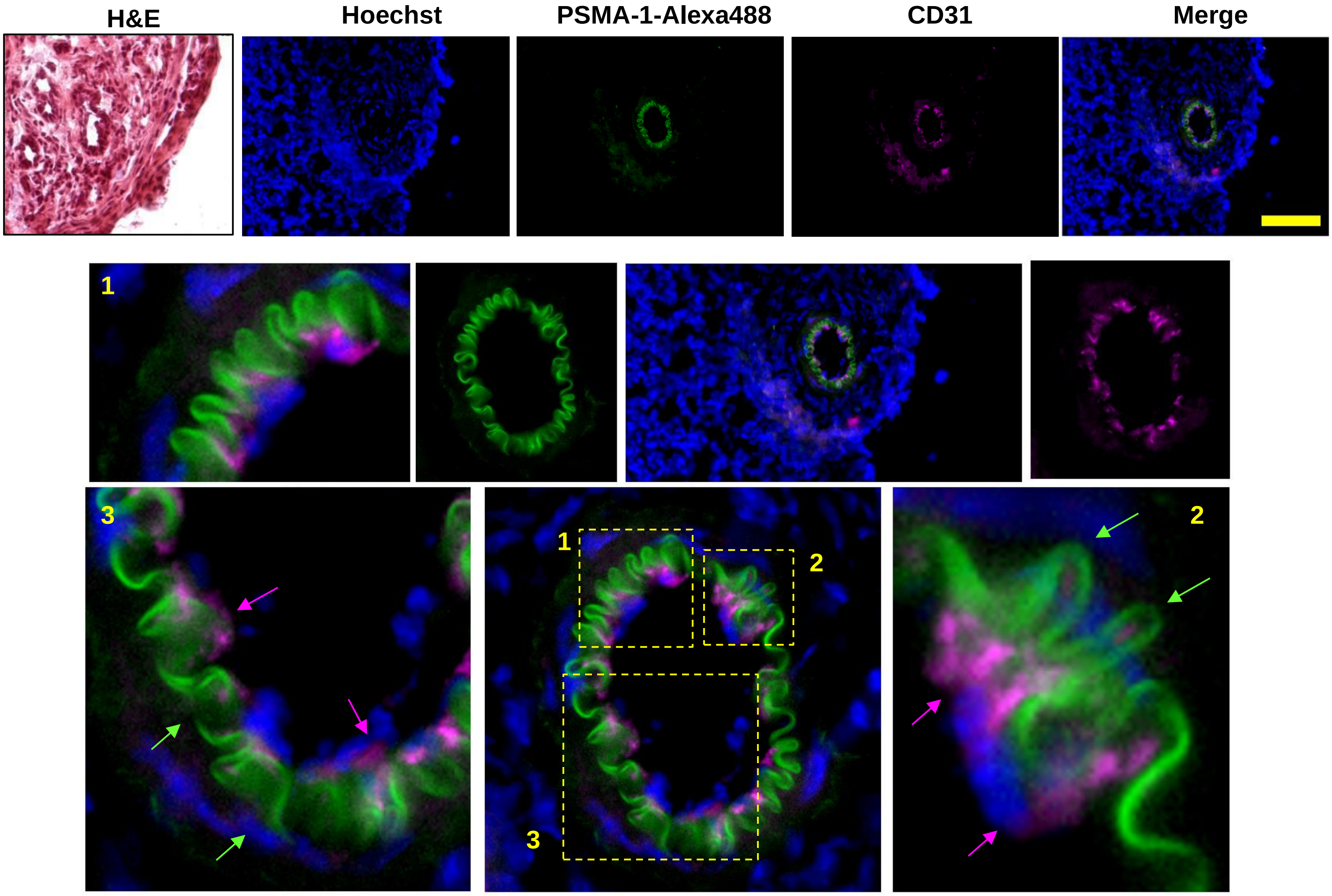


**Fig. S8:** Additional images of PSMA-CD31 colocalization in 4T1 tumors from immunocompetent BALB/c mice, measurement bar signifies 100 μm. We can see PSMA expression within the neovasculature. PSMA shows a distinct staining pattern here which may be caused due to the structure of the inner luminal walls of the neovasculature.

**Fig.S9: Validation of PSMA-1-Alexa488 Staining for PSMA Through The Use of Commercial PSMA Primary Antibody + AlexaFluor 594 Secondary Antibody in Adjacent 4T1 Primary Tumor Sections**

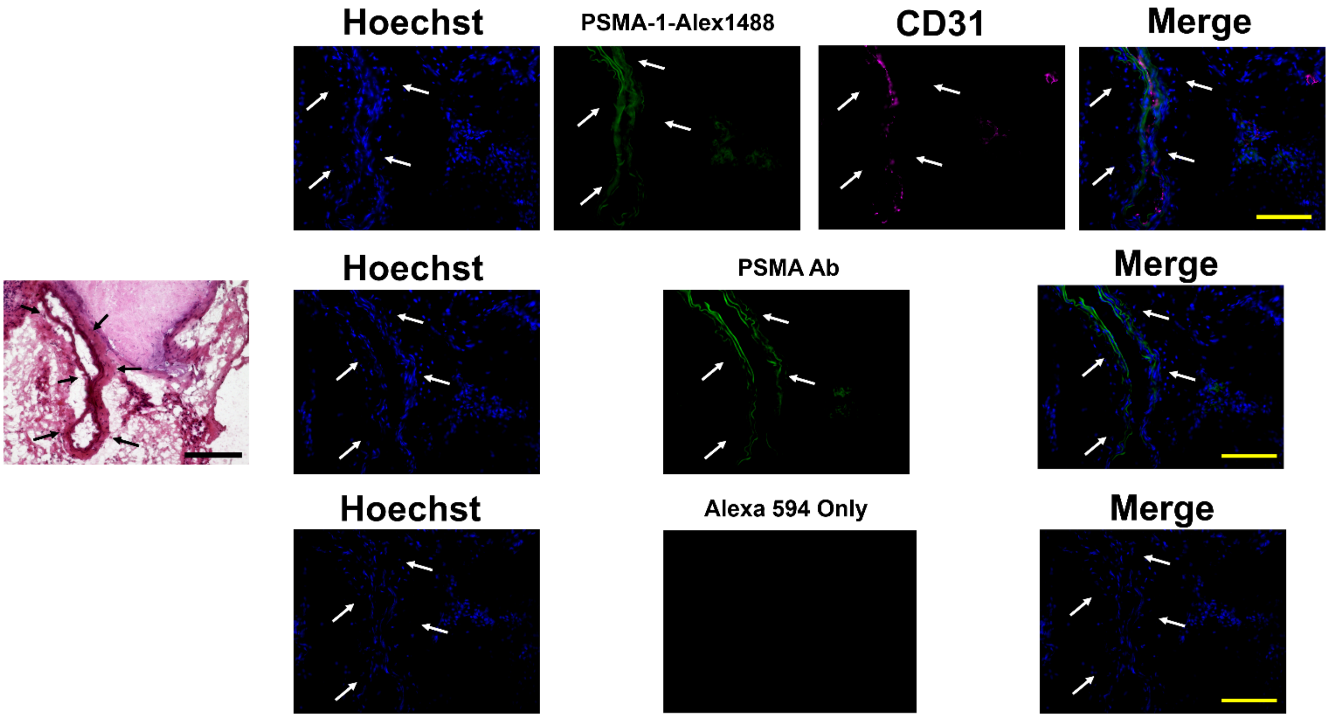


**Fig. S9:** Stains of adjacent 4T1 primary tumor tissues. The first row shows staining using PSMA-1-Alexa488, developed in our lab, co-stained with CD31 primary antibody and AlexaFluor 594 secondary antibody. The second row shows staining using commercially available PSMA primary antibody (PSMA Ab). The third row shows staining with AlexaFluor 594 secondary antibody only. This example validates the expression of PSMA within these tissues as well as the specificity of our PSMA-1 ligand as targeting moiety. Scale bar represents 100 μm and it applies to all images.

**Fig.S10 - PSMA Expression on Cells and not Neovasculature in PSMA Expressing Prostate Cancer**

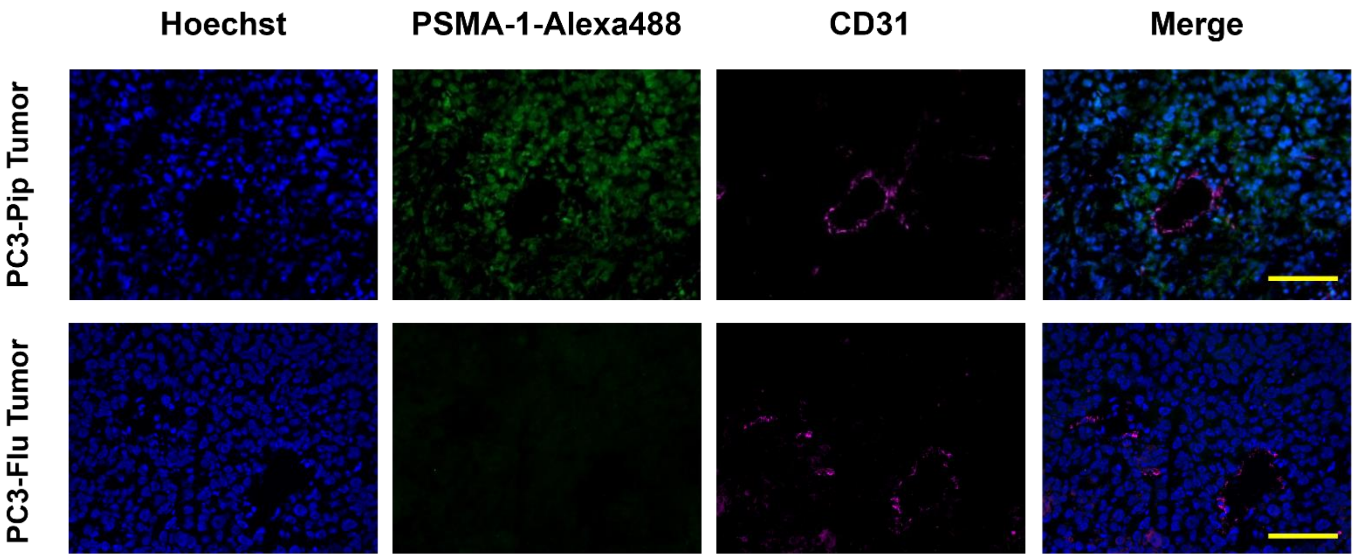


**Fig.S10:** PSMA-CD31 staining in PC3-Pip (high PSMA expression) and PC3-Flu (little to no PSMA expression) prostate cancer tumors. We can see the expression of PSMA on the cells themselves instead of the neovasculature as with our breast cancer models. Scale bars represent 100 μm.

**Fig.S11: Example of PSMA-CD31 PCC and MC Values in Various Tissues**

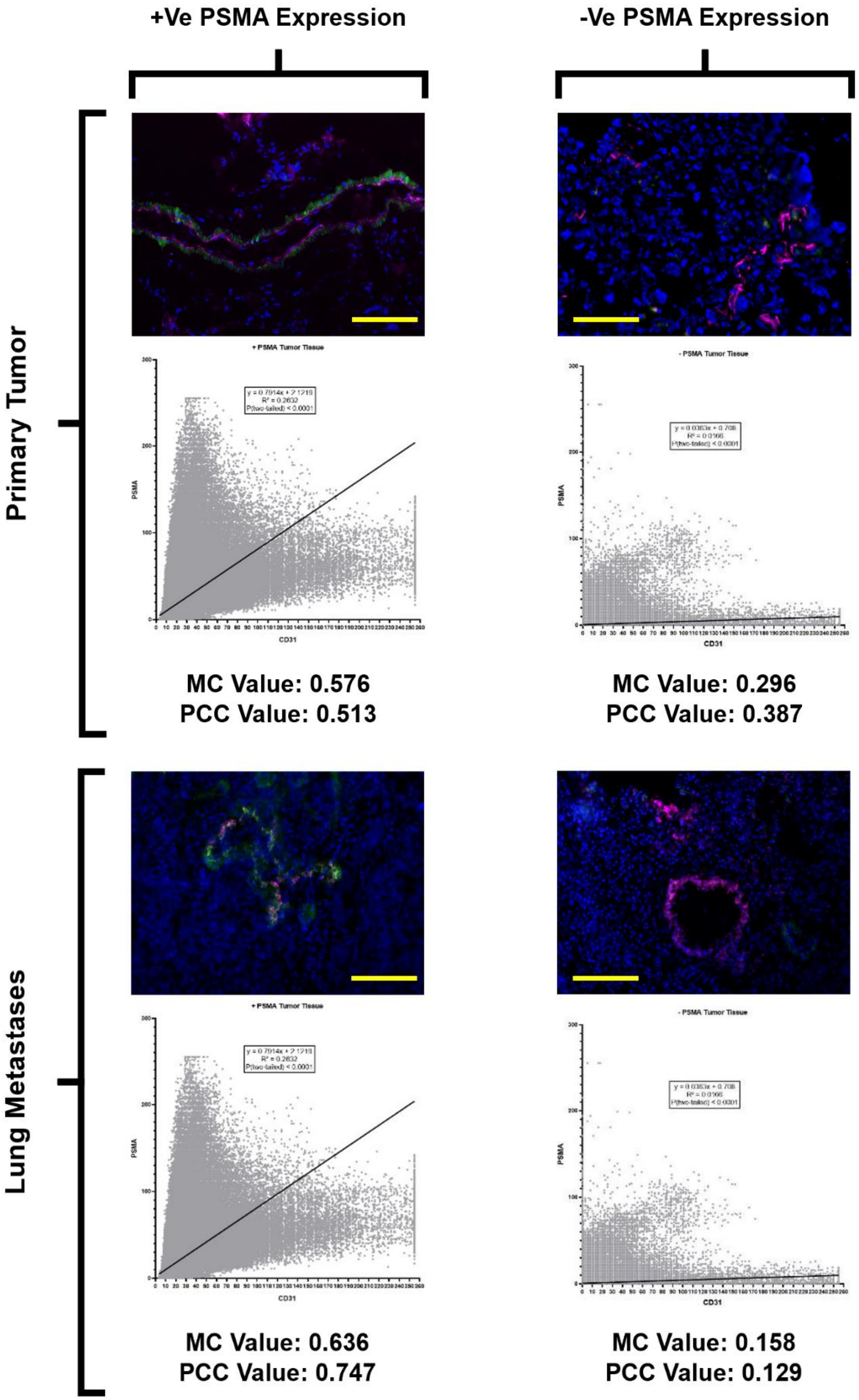

**Fig. S11:** Examples of areas with positive PSMA and little/no PSMA expression in both primary tumor and lung metastases tissues with corresponding cytofluorogram and MC and PCC values and the PSMA (green) to CD31 (magenta) intensities pertaining to these samples. In this method, whole tissues were initially cut into 5 sections with 3 pieces of tissues from each of the five sections. Yellow Bars measure 100μm.

**Fig.S12: Validation of PSMA-1-Alexa488 Staining for PSMA Through The Use of Commercial PSMA Primary Antibody + AlexaFluor 594 Secondary Antibody in Adjacent 4T1 Lung Metastases tissues**

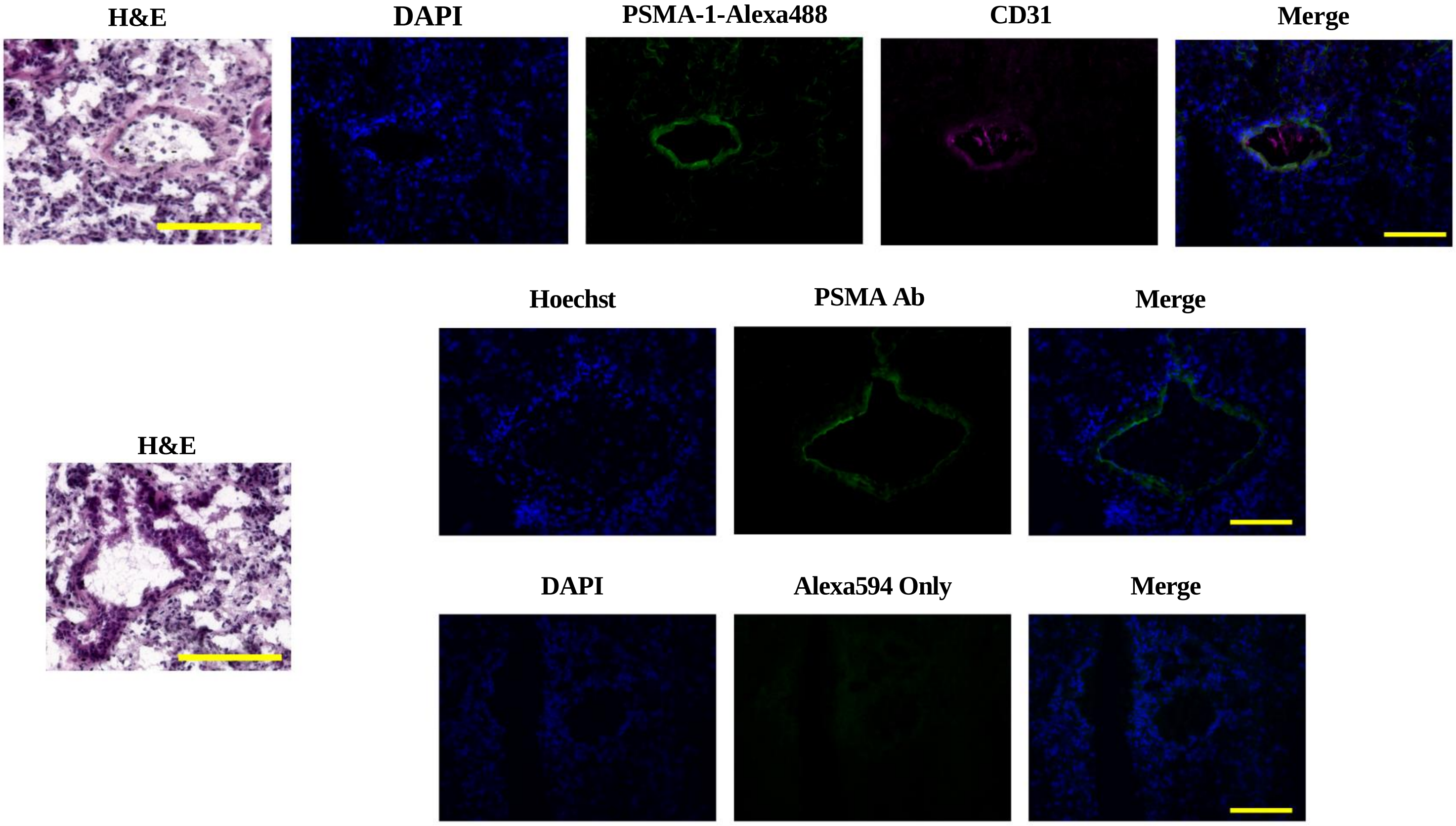


**Fig. S12:** Stains of adjacent 4T1 lung metastases tissues. Scale bars represent 100µm. The first row shows staining using PSMA-1-Alexa488, developed in our lab, co-stained with CD31 primary antibody and AlexaFluor 594 secondary antibody. The second row shows staining using commercially available PSMA primary antibody (PSMA Ab). The third row shows staining with AlexaFluor 594 secondary antibody only. "Merge" with PSMA-only staining reveals CD31 specificity, confirming that the exclusion of primary Abs provides for accurate results and no false positives. "This example validates the expression of PSMA within these tissues as well as the specificity of our PSMA-1 ligand as targeting moiety.

**Fig.S13: PSMA-Pc413 Accumulation in M4 and M5 from Fig. 6**

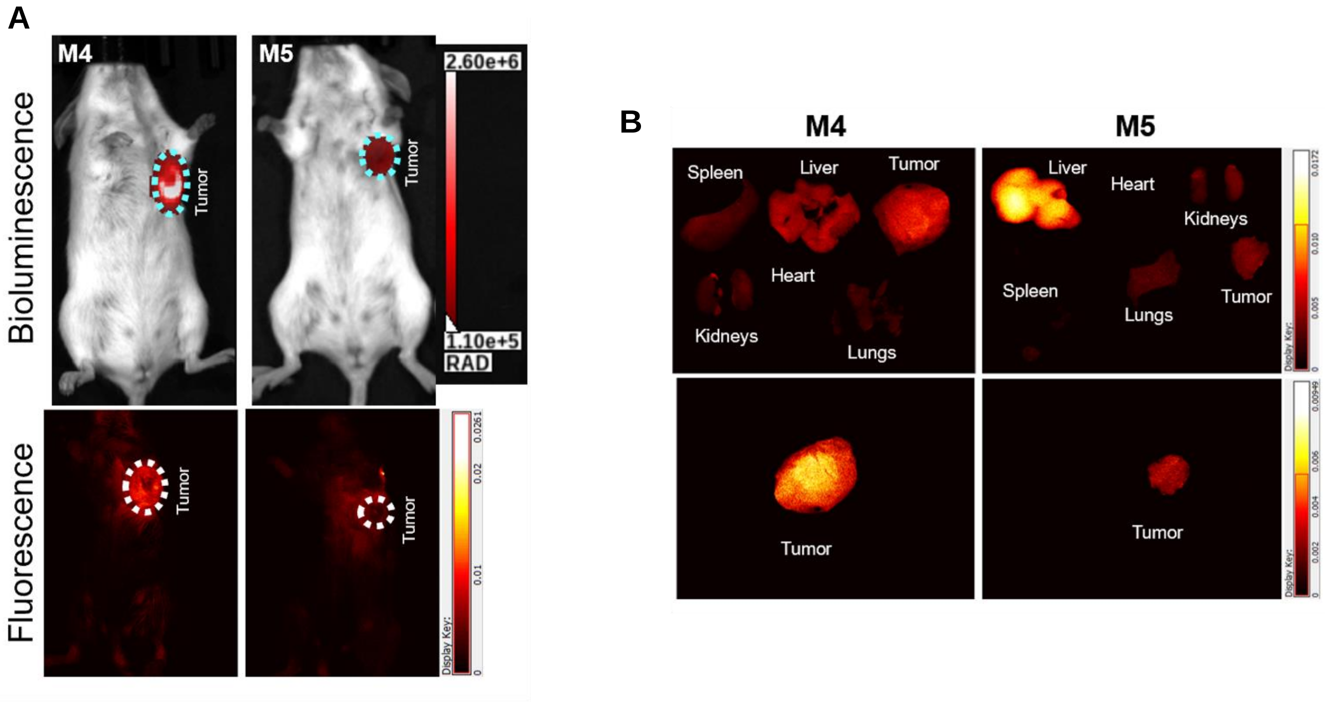


**Fig.S13: (A)** Fluorescence and Bioluminescence imaging of two of five mice that received PSMA-1-Pc413 not presented in **Fig. 6**. These mice had less advanced tumors which had not yet metastasized to the lungs as can be seen with minimal PSMA-1-Pc413 accumulation in the lungs and around the body. These signals were averaged with M1-M3 seen in the main text in **Fig. 6B.** M5 showed reduced PSMA-1-Pc413 and bioluminescence signal and had a much small primary tumor, however, both imaging modalities were able to differentiate the tumor tissue compared to normal structures. Please note fluorescence images are on the same scale as M1-M3 in the amin text. **(B)** Fluorescence signal on extraction of the organs. While M5 showed higher fluorescence intensity from the liver, this is the organ of excretion of PSMA-1-Pc413. However, on imaging the tumor separately we can still see significant uptake in tumor. Tumor to background ratio (TBR) values: M4 Tumor 1.67; M5 Tumor 1.04.

**Fig.S14: PSMA-Pc413 Accumulation Over Time in 4T1 Tumors in BALB/c Mice**

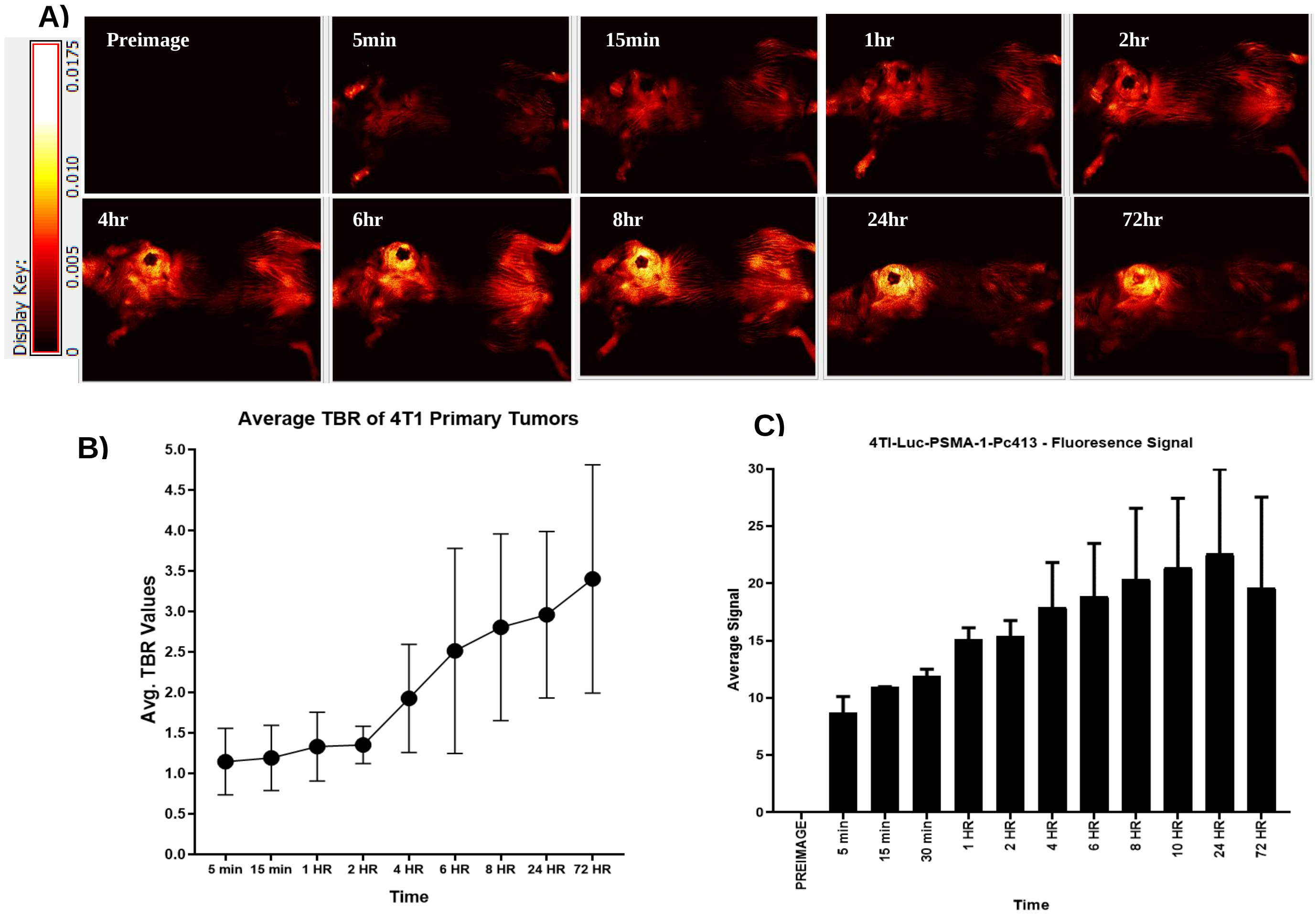


**Fig.S14(A):** Imaging of PSMA-1-Pc413 in a BALB/c mouse inoculated with 4T1 tumor in the top right breast pad. The representative mice imaged here has a scab on the tumor surface observed as a dark spot. **(B)** The average TBR values for M1-5 have been plotted across time by measuring tumor signal and abdominal skin/fur as background. We can see TBR values increasing over time till 72hr. TBR > 1.5 means tumor tissues can be distinguished from normal structures. In this study, TBR > 1.5 between 2-4hrs and continues out to 72hr. **(C)** Average of the fluorescence signal in the tumor of five mice over time. We observe an increase in PSMA-1-Pc413 fluorescence signal till 24hrs after which we see the signal decrease as the probe clears. Signals have been normalized to the background.

**Fig.S15: PSMA-Pc413 Accumulation In Various Tumors 24hrs post PSMA-1-Pc413 injection**

**A) Fluorescence Signal at 24hr Post IV Injection of PSMA-1-Pc413**

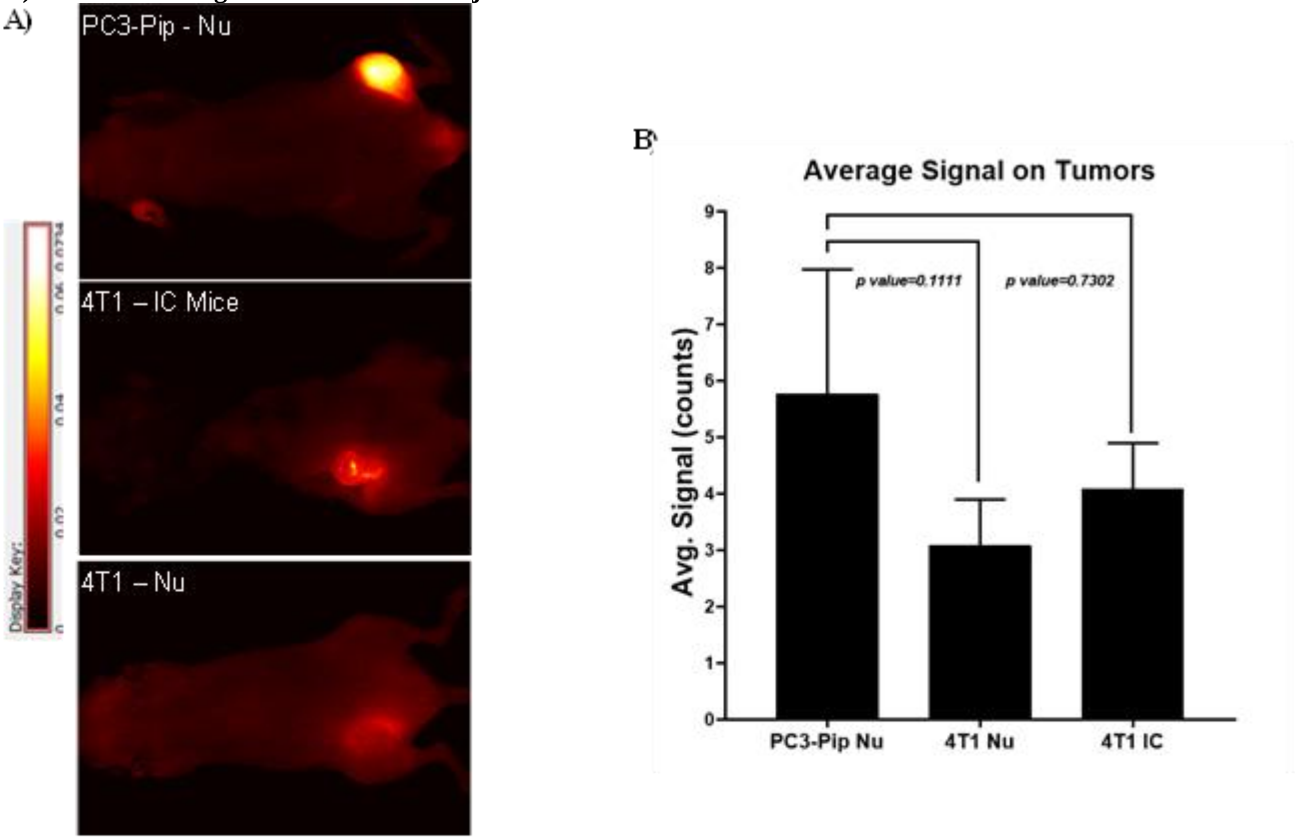


**Fig.S15: Nu = Immunocompromised mice, IC = Immunocompetent mice. (A)** We wanted to compare PSMA-1-Pc413 uptake in prostate cancer and breast cancer in both BALB/c and Nude mice. 5 athymic, male mice were inoculated with PSMA expressing PC3-Pip tumors in the right flank; 5 female, BALB/c mice were inoculated with 4T1 tumors in the bottom left mammary pad, and lastly, 5 female, athymic mice were inoculated in the bottom left mammary pad. 0.5mg/kg of PSMA-1-Pc413 was administered via tail vein injection to all mice. **(B)** The average signal at peak accumulations (at 24hrs) are shown with uptake being half of PC3Pip Prostate tumors. We can observe no significant difference between PC3-Pip tumors and both athymic and immunocompetent 4T1 groups. $p_{4T1\ Nu} = 0.111 > 0.05$ and is therefore not significant; $p_{4T1\ IC} = 0.730 > 0.05$ and is therefore not significant.
